# Leveraging target enrichment and genome skimming (Hyb‐Seq) of herbarium collections to unlock timber DNA barcoding

**DOI:** 10.1002/aps3.70063

**Published:** 2026-06-12

**Authors:** Sidonie Bellot, Dyana Ndiade Bourobou, Camila Quintero‐Berns, Laszlo Csiba, Peter Gasson, Barrett McBride, Bhely Angoboy Ilondea, Gaël U. D. Bouka, Emmanuel Ebanyenle, Cynel Gwenael Moundounga, Martin Ricker, Philomena Yarwoah, Guy Herman Zanguim Tchoutezou, Janvier Lisingo, Victor Deklerck

**Affiliations:** ^1^ Royal Botanic Gardens Kew, Richmond TW9 3AE United Kingdom; ^2^ Institut des Recherches Agronomiques et Forestières Centre National de la Recherche Scientifique et Technologique (IRAF‐CENAREST) BP 2246, Campus de Gros‐Bouquet Libreville Gabon; ^3^ University of Reading Reading Berkshire United Kingdom; ^4^ Institut National pour l'Etude et la Recherche Agronomiques 13 Avenue des Cliniques BP 2037 Kinshasa/Gombe Democratic Republic of the Congo; ^5^ Université Pédagogique Nationale (UPN) BP 8815 Kinshasa‐Ngaliema Democratic Republic of the Congo; ^6^ Biodiversity Laboratory, Ecosystems and Environment Management, Faculties of Science and Technology Marien Ngouabi University BP 69 Brazzaville Republic of Congo; ^7^ CSIR‐Forestry Research Institute of Ghana UPO 63 Kumasi Ghana; ^8^ Institut des Recherches en Ecologie Tropicale Centre National de la Recherche Scientifique et Technologique (IRET‐CENAREST) Libreville Gabon; ^9^ Departamento de Botánica, Instituto de Biología Universidad Nacional Autónoma de México (UNAM) Ciudad de México Mexico; ^10^ National Herbarium of Liberia Forestry Training Institute Tubmanburg Liberia; ^11^ Department of Geography, Environment and Geomatics University of Ottawa 75 Laurier Ave. E Ottawa K1N 6N5 Ontario Canada; ^12^ Department of Forestry University of Dschang BP 96 Dschang Cameroon; ^13^ Laboratoire d'écologie et aménagement forestier, Faculté des sciences, University of Kisangani Democratic Republic of the Congo; ^14^ Meise Botanic Garden, Meise Belgium

**Keywords:** Angiosperms353, *Entandrophragma*, ITS, *Khaya*, *Lovoa*, *Swietenia*, *trnL‐trnF*, wood DNA extraction

## Abstract

**Premise:**

DNA barcoding for timber species identification requires comprehensive reference datasets, informative DNA barcodes, and cost‐effective protocols. We developed a workflow leveraging Hyb‐Seq (target capture sequencing and genome skimming) to address these challenges, and we tested it on four genera from the mahogany family (Meliaceae).

**Methods:**

We sequenced up to 350 nuclear and 177 plastid loci from 132 herbarium specimens representing leaf samples of 22 species. We determined the DNA barcoding potential of each locus by looking at species recovery and monophyly in gene trees. We then selected 13 short regions (candidate barcodes) within high‐potential loci and tested their PCR amplification and Sanger sequencing on wood DNA.

**Results:**

Three candidate barcodes emerged as the most reliably sequenced from wood DNA and as providing the most accurate species‐level identifications, with species monophyly rates above 80%. Failure to obtain sequences from some wood DNA extracts was more often associated with potential DNA impurity (as inferred from DNA color) than with DNA degradation.

**Discussion:**

Our reference data and candidate barcodes provide a foundation to support the DNA barcoding of mahogany and its relatives. Our workflow illustrates how the wealth of Hyb‐Seq data currently generated from global herbaria may be leveraged to monitor plant diversity.

The illegal timber trade represents a major environmental crime, with 50–90% of the timber originating from key producer countries being illegally logged (INTERPOL/World Bank, 2009). Illegal logging has negative impacts on people, ecosystems, and economies (Pacheco et al., 2016), and mechanisms have been implemented to control it, from national traceability systems to multilateral treaties (Momballa‐Mbun et al., 2023). Successful timber monitoring relies on the ability to identify species at strategic points of the supply chains (Lowe et al., 2016), including when leaves, flowers, and bark have been removed. Species‐level identification using DNA, i.e., DNA barcoding (Hebert et al., 2003), can be highly powerful in such cases where the removal of morphological features hinders sample identification and tracking. For instance, DNA barcoding has been used successfully to identify wood from various plant families (Nithaniyal et al., 2014; Jiao et al., 2019; But et al., 2023), to demonstrate the illegal logging of timber in the United States (Lowe et al., 2016) and the poaching of cycads in South Africa (Williamson et al., 2016), and to authenticate herbal supplements (de Boer et al., 2015). This approach has therefore been suggested as a tool to monitor the illegal timber trade (Dormontt et al., 2015); however, the application of DNA barcoding to timber species identification remains restricted due to technical challenges.

A first challenge is that the accurate DNA‐based identification of a sample requires the comparison of a region of its genome (i.e., the “barcode”) to a reference dataset comprising sequences of the same DNA barcode from all species to which the sample could possibly belong (Hebert et al., 2003). The representativeness of the reference is crucial to prevent the wrong species name being assigned to the sample. To accommodate intraspecific genetic variation that could result in wrong identification (Meyer and Paulay, 2005), the reference should also ideally contain multiple representatives of each species from throughout the species’ range. However, even comprehensive repositories of plant DNA barcoding data such as the Barcode of Life Database (BOLD; Ratnasingham and Hebert, 2007) lack multi‐species, multi‐individual reference DNA datasets for many timber lineages. A second challenge is the lack of DNA barcodes that are suitable for species‐level identification of timber species. Standard plant DNA barcodes, including the nuclear ribosomal ITS regions and plastid regions such as *rbcL*, *matK*, *trnH*‐*psbA*, or the *trnL* intron (Hollingsworth et al., 2016), can be sequenced by PCR and Sanger sequencing from many plant lineages using published “universal” primers, removing the need to design new barcodes and primers for specific genera. However, these standard barcodes do not always vary sufficiently to distinguish species, even when combined (CBOL Plant Working Group, 2009; Jones et al., 2021), and previous studies have highlighted the need for more variable barcodes in many timber lineages (Muellner et al., 2011; Parmentier et al., 2013; Hassold et al., 2016). Moreover, although such DNA barcoding approaches based on the PCR of a few barcodes tend to be simpler, quicker, and cheaper to put in place than approaches relying on high‐throughput sequencing, their use for timber DNA barcoding raises a third challenge. Indeed, wood DNA is often degraded and contaminated by plant metabolites, which can lead to PCR failure (Jiao et al., 2020). Although previous studies have had success in amplifying and sequencing standard barcodes from wood dust (Kannangara et al., 2020) or from fresh or dried heartwood (Jiao et al., 2014), the relatively large size of these barcodes often prevents their amplification from the highly degraded DNA found in processed or aged wood (Jiao et al., 2014, 2020). This requires the development of additional primers to sequence the barcodes in multiple steps (Höltken et al., 2012; Tanaka and Ito, 2020) or the development of new, shorter barcodes. As a result, barcodes that are informative but short enough to be PCR‐amplified from degraded wood DNA are yet to be found for many timber lineages (Lowe and Cross, 2011; Jiao et al., 2020). In this context, developing a workflow to generate comprehensive reference data, identify new DNA barcodes, and test their PCR amplification from wood could unlock timber DNA barcoding applications for many plant groups.

Among the plant lineages that could benefit from such a workflow are four Meliaceae genera comprising high‐value African and American timber species: *Entandrophragma* C.DC. (11 species), *Khaya* A.Juss. (eight species), *Lovoa* Harms (two species), and *Swietenia* Jacq. (three species). *Khaya* species are referred to as acajou, while *Swietenia* species are known as mahogany (Ward et al., 2008; Bouka et al., 2019). These species and look‐alikes from the African genus *Entandrophragma* are often intermingled in timber supply chains, which hinders the control of their trade (Gasson, 2011; Bouka et al., 2019; Deklerck et al., 2019). Due to their intense exploitation, all *Swietenia* species, five *Khaya* species, and six *Entandrophragma* species are assessed as “Vulnerable,” “Endangered,” or “Near Threatened” on the International Union for the Conservation of Nature (IUCN)'s Red List of Threatened Species (IUCN, 2024). Although *Lovoa* species are not mahogany look‐alikes, they are closely related to the other genera, and the monitoring of *Lovoa trichilioides* Harms (known as dibetou) is recommended to avoid population declines resulting from overexploitation (Barstow, 2018). To strengthen their protection, all *Khaya* and *Swietenia* species have been listed in Appendix II of the Convention on International Trade in Endangered Species of Wild Fauna and Flora (CITES); however, none of the *Entandrophragma* or *Lovoa* species are listed yet. Sustainable management of these species is essential to avoid their loss from forest ecosystems and local economies, where they are valued for the food or medicinal compounds that they provide (Tieguhong and Ndoye, 2007; Louppe et al., 2008; Lisingo et al., 2010; Yadav et al., 2015).

DNA barcoding has already been explored as a potential route towards improving the monitoring of these species. Standard DNA barcodes, especially ITS and the whole plastid genome, have shown good species‐level discrimination for *Entandrophragma* but are less effective for *Swietenia* and *Khaya* (Muellner et al., 2003, 2011; Monthe et al., 2019; Mascarello et al., 2021; Bogun et al., 2024). Efforts have also been made to look beyond standard barcodes using genome skimming or restriction site–associated DNA sequencing (RAD‐seq) data. As a result, a set of just 15 (and up to 101) nuclear, plastid, and mitochondrial single‐nucleotide polymorphisms (SNPs) has been found to provide high species‐level resolution in *Khaya* (Pakull et al., 2016, 2019; Bouka et al., 2022), while a set of 120 species‐specific SNPs were identified in *Swietenia* (Pakull et al., 2020). Although these studies included a few DNA samples extracted from wood (mainly cambium), they did not report success rates for this type of sample. An early exception is Höltken et al. (2012), who successfully characterized at least one plastid SNP from degraded wood DNA, but the marker they studied only enables genus‐level identification. There are currently no go‐to barcodes and protocols that can be used when wood samples that may belong to any of these genera need to be identified to species level.

The advent of Hyb‐Seq approaches enables the screening of hundreds of plastid and nuclear genetic regions from non‐model species by combining genome skimming and target capture sequencing (Weitemier et al., 2014). These approaches can facilitate the development of a workflow to design and test new DNA barcodes by offering solutions to the three above‐mentioned challenges surrounding timber DNA barcoding. Indeed, Hyb‐Seq approaches using short‐read sequencing can accommodate the degraded DNA found in herbarium specimens, making it suitable for the generation of comprehensive reference genomic datasets from historical collections. Moreover, the Angiosperms353 target capture probe kit enables the sequencing of 353 genetic regions across all angiosperms (Johnson et al., 2019) that are phylogenetically informative at the genus and species levels (Baker et al., 2021; Slimp et al., 2021). This suggests that reference datasets including Angiosperms353 regions could be used to identify new DNA barcodes or to validate the discrimination power of existing barcodes (Albreht et al., 2025). Crucially, this process could be designed to identify barcodes amenable to PCR and Sanger sequencing from degraded DNA such as that found in wood. Despite the wide uptake of Hyb‐Seq by phylogenomic studies, its potential to support the development of DNA barcoding resources remains largely untapped, especially in the context of timber monitoring.

Here, we develop and test a workflow to design a DNA barcoding toolkit comprising Hyb‐Seq–based reference data and DNA barcodes suitable for species‐level identification of wood DNA through PCR and Sanger sequencing (Figure 1). This workflow involves: (1) building a well‐resolved multi‐gene, multi‐species, multi‐individual reference phylogenetic framework based on genome skimming and target capture sequencing data; (2) identifying a subset of genes with high barcoding and low paralogy potential by analyzing reference sample recovery and species monophyly in gene trees, as well as the number of assembled copies for each gene; (3) defining short yet informative candidate barcodes in some of the genes with the highest potential for barcoding and designing corresponding PCR primers; and (4) testing which of the candidate barcodes provide the best combination of Sanger sequencing success from wood DNA and species‐level identification success (Figure 1). The latter step also involves an optional comparison of the lab performance and discriminatory power of the new barcodes with those of selected standard barcodes. Illustrating the power of this workflow, we found that applying it to *Entandrophragma*, *Khaya*, *Lovoa*, and *Swietenia* yielded insights into their evolutionary relationships and enabled the design of new candidate DNA barcodes that could support species‐level identification of their timber.

**Figure 1:**
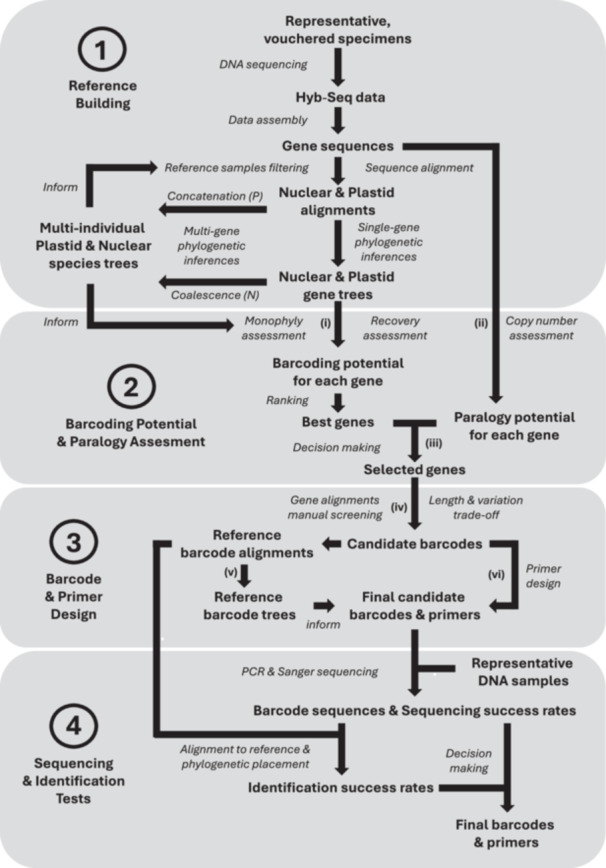
Workflow for the Hyb‐Seq–based development of reference data and DNA barcodes suitable for species‐level identification of wood DNA through PCR and Sanger sequencing. The workflow consists of four main steps (numbered from 1 to 4, described in the Introduction), and the barcode search and design (steps 2 and 3) consist of six substeps (i to vi, described in the Methods section).

## METHODS

The description of the Methods follows the workflow presented in Figure 1. Details are provided in the supplementary methods provided in Appendix S1 (see Supporting Information with this article).

### Reference building

To build a reference dataset (box 1 of Figure 1), we generated Hyb‐Seq data from herbarium specimen leaf tissue sampled in Kew Herbarium and in World Forest ID collections held at Kew (https://worldforestid.org; Appendix 1). We aimed to sample individuals from across the native distribution range of each species to maximize the genetic variation and representativity of our dataset. As a result, our final reference DNA dataset comprises data for all 11 *Entandrophragma*, two *Lovoa*, and three *Swietenia* species, and all but one (*K. madagascariensis* Jum. & H.Perrier) of the eight *Khaya* species, with each species represented by two to 18 individuals from multiple countries, except for *E. bussei* Harms ex Engl., *E. palustre* Staner, and *K. euryphylla* Harms, for which only one individual each could be sourced. We also added 33 wood samples to the Hyb‐Seq workflow described below, not to be included in the reference, but to test if they could be identified using this approach. All samples are listed in Appendix S2 with their geographic origin and voucher information, and details on the wood samples and on wood‐specific modifications of the protocols described below are provided in Appendix S1.

DNA extraction was performed using a modified cetyltrimethylammonium bromide (CTAB) method (Doyle and Doyle, 1987) described in Brewer et al. (2019), and DNA libraries were prepared with a NEBNext Ultra II library kit (New England BioLabs, Hitchin, United Kingdom). Library aliquots were submitted to target sequence capture with the Angiosperms353 kit (Johnson et al., 2019) manufactured by Daicel Arbor Biosciences (Ann Arbor, Michigan, USA), following their standard protocol (https://arborbiosci.com/wp-content/uploads/2023/06/myBaits_Manual_v5.03.pdf). Enriched and non‐enriched library fractions were then sequenced on an Illumina NovaSeq X platform (Illumina, San Diego, California, USA) at Macrogen (Seoul, Korea) to generate 150‐bp‐long paired‐end sequencing reads. Angiosperms353 target sequence capture data of *Schmardaea microphylla* H.Karst. ex Müll.Berol. (accession ERR7620407; Baker et al., 2022) were retrieved from the European Nucleotide Archive so that this species could be used as outgroup.

Target capture sequencing and genome skimming data were pooled together, their quality was assessed using FASTQC v.0.11.9 (Andrews, 2010), and adapters and low‐quality bases were removed using Trimmomatic v.0.39 (Bolger et al., 2014). After preliminary analyses using HybPiper v.2 (Johnson et al., 2016), we used CAPTUS v.1 (Raza et al., 2023; Ortiz et al., 2024) to perform the final assembly of the Angiosperms353 regions, the ITS region, and 177 plastid regions (see Appendix S1 for details on target regions and the reference files used to assemble them). The CAPTUS analysis and downstream steps were performed with and without excluding the four samples assigned to *K. anthotheca* C.DC., as they were in fact of dubious identity (Appendix S1; Discussion). This allowed us to keep these samples in reference alignments used for barcode search while excluding them from the tree presented in the Results section.

We compared two approaches for handling paralogy in downstream phylogenetic analyses: a paralog‐inclusive (PI) approach using all copies (potential paralogs) assembled for a given gene, and a paralog‐exclusive (PE) approach using only one of the copies (selected by CAPTUS based on its similarity to the reference sequence). For each nuclear (including ITS) and plastid region, the sequences of all samples (including potential paralogs in the PI analysis) were aligned using MAFFT v.7 (Katoh and Standley, 2013) and the alignments were cleaned using ClipKIT (Steenwyk et al., 2020) inside the CAPTUS pipeline, CIAlign v.1.1.0 (Tumescheit et al., 2022), and TAPER v.1.0 (Zhang et al., 2021). Clean nuclear alignments with a median ungapped sequence length <300 bp were discarded as they would likely not contain enough phylogenetic signal to inform gene tree inferences, while all plastid alignments were kept to be concatenated. This resulted in 350 (PE approach) or 343 (PI approach) Angiosperms353, one ITS, and 177 plastid clean alignments.

For each nuclear alignment, a gene tree was estimated with IQ‐TREE v.1.6.12 (Minh et al., 2020), implementing ModelFinder (Kalyaanamoorthy et al., 2017) and 1000 ultrafast bootstrap replicates. The nuclear gene trees resulting from the PE approach (except the ITS tree, which was kept separate) were analyzed using weighted ASTRAL v.1.16.3.4 (Zhang et al., 2018; Zhang and Mirarab, 2022) to generate a species tree. The nuclear gene trees resulting from the PI approach (and therefore often comprising multiple gene copies for a given sample) were decomposed into single‐copy gene trees using DISCO v.1.4 (Willson et al., 2022), and the latter were then analyzed with weighted ASTRAL to generate a species tree where each sample was represented only once. A concatenated matrix of all plastid alignments was made with AMAS (Borowiec, 2016), and this was provided to IQ‐TREE for phylogenetic inference with 1000 bootstrap replicates and best partition scheme and nucleotide substitution model searches using ModelFinder and the TESTMERGE option (Chernomor et al., 2016).

### Barcoding potential and paralogy assessment

To identify genes in which suitable barcodes might be found, we analyzed gene trees and gene alignments in three steps (steps i to iii; box 2 of Figure 1): (i) the barcoding potential of each gene is assessed, based on sample recovery and species monophyly in gene trees; (ii) the risk of paralogy is assessed for genes with a barcoding potential higher than a user‐defined threshold; and (iii) genes satisfying user‐defined requirements of barcoding and paralogy potentials are selected for barcode search.

For step i (barcoding potential assessment), a custom R script was used to assess the barcoding potential of each gene based on two criteria. The first criterion was the rate of recovery of the gene among our samples. This criterion was chosen because a better recovery would enable a more informed primer design. The rate of recovery of a gene was obtained by calculating the percentage of individuals of each species present in the gene tree and averaging this percentage across species. The second criterion was the rate of monophyly. This criterion reflects how the information contained by the gene allows samples of different species to be distinguished from each other. The rate of monophyly of a gene was obtained by calculating the percentage of individuals of each species present in the largest clade made by individuals of this species in the gene tree and then averaging this percentage across species. R scripts and input gene trees are available at https://github.com/sidonieB/Bellot_al_Meliaceae_DNA_barcoding (see Data Availability Statement). Visualizations and analyses of species and gene trees were performed using the packages ape v.5.0 (Paradis and Schliep, 2019), dplyr v.1.1.4 (Wickham et al., 2023), ggrepel v.0.9.6 (Slowikowski, 2024), ggtree v.3.10.1 (Xu et al., 2022), ggplot2 v.3.5.1 (Wickham, 2016), gridExtra v.2.3 (Auguie, 2017), phytools v.2.3.0 (Revell, 2012), plyr v.1.8.8 (Wickham, 2011), reshape2 v.1.4.4 (Wickham, 2007), tidyr v.1.3.1 (Wickham et al., 2024), and treeio v.1.26.0 (Wang et al., 2020; Yu, 2022) in R 4.3.0 (R Core Team, 2023) and RStudio 2024.04.2 (Posit Team, 2024).

The second step (paralogy assessment) was only performed on genes that had a high barcoding potential based on the above two criteria, i.e., those that had both a rate of recovery and a rate of monophyly superior to 70%. The presence of multiple copies may prevent cloning‐free PCR and Sanger sequencing and result in misleading identification results, so the potential for paralogy of a gene was defined as the number of samples having more than one copy of the gene according to the statistics provided by CAPTUS. For step iii (gene selection), we selected four genes with different degrees of barcoding and paralogy potential (see Results) in order to explore how our estimations of barcoding and paralogy potentials affected barcode success in practice.

### Barcode and primer design

To identify candidate barcodes and design primers for the PCR amplification and Sanger sequencing, we analyzed the four selected genes in three additional steps (steps iv to vi; box 3 of Figure 1): (iv) the sequence alignments of the selected genes are examined to select small variable regions (i.e., the candidate barcodes), (v) phylogenetic trees are generated for each candidate barcode to assess their ability to discriminate between species and optionally rule out some candidates, and (vi) primers are designed in conserved regions surrounding the final candidate barcodes.

For step iv (candidate barcode search), the untrimmed alignments of the selected genes were obtained from CAPTUS and screened using Geneious Prime 2024 or UGENE v.52 (Okonechnikov et al., 2012) to look for relatively short but variable regions that could serve as barcodes, flanked by more conserved regions in which PCR primers could be designed that would likely work across genera. We aimed to find regions between 150 bp and 400 bp long with as much variation as possible between species. This length was chosen based on the range of DNA sizes that we obtained from wood samples (see Results), as this would increase the chance that the barcodes could be amplified by PCR from such samples. This resulted in the selection of 13 candidate barcodes.

For step v (barcode trees), candidate barcode regions were extracted from the reference gene alignments and further cleaned by removing sequences from wood samples and a few highly divergent sequences (likely to result from misassembly). They were then submitted to phylogenetic inference using IQ‐TREE with automatic selection of the nucleotide substitution model and 1000 ultrafast bootstrap replicates (Kalyaanamoorthy et al., 2017; Minh et al., 2020). Three additional trees were inferred from concatenated alignments of some of the barcodes (see Results) to evaluate whether selected barcode combinations could increase phylogenetic resolution. The barcode reference alignments and trees are available at https://github.com/sidonieB/Bellot_al_Meliaceae_DNA_barcoding. Although we used the barcode trees to discuss which candidate barcodes and barcode combinations were the best (see Results and Discussion), we did not use them to rule out barcodes at this step as we wanted to test all barcodes in the lab. Primer pairs were then designed (step vi) for the 13 barcodes using Primer3Plus (Untergasser et al., 2012). Primer design failed for one of the regions (which we called 5816_r3) because of overlooked sequence variation, so this region was not included in the study, resulting in 12 final candidate barcodes.

### Sequencing and identification tests

The PCR amplification and Sanger sequencing of the 12 candidate barcodes were tested on 10 sapwood and nine heartwood DNA extracts from six species of commercial importance representing the four focus genera, namely *E. cylindricum* Sprague, *E. candollei* Harms, *K. ivorensis* A.Chev., *K. senegalensis* (Desr.) A.Juss., *L. trichilioides*, and *S. macrophylla* King in Hook. To evaluate how our candidate barcodes behaved compared with widely used plant genetic markers, we also tested the PCR and Sanger sequencing of the ITS1 region and of the plastid intergenic spacer *trnL‐trnF* on the same DNA extracts. PCR was performed using positive and negative controls, and PCR products were analyzed by electrophoresis and purified using the NucleoSpin Purification Kit (Macherey‐Nagel, Düren, Germany). Clean products were sequenced using a 3730xl DNA Analyzer (Applied Biosystems, Waltham, Massachusetts, USA). The list of samples used is provided in Appendix S3 with indications on how the DNA was extracted for each sample, while the full DNA extraction and PCR protocols are provided in Appendix S1.

Barcode sequences were cleaned using Geneious Prime 2024 or UGENE (Okonechnikov et al., 2012), combining forward and reverse reads when both were available and checking for non‐plant contamination by matching them against the Core Nucleotide Database of the National Center for Biotechnology Information's GenBank. The sequences were then aligned to the reference sequences available for each candidate barcode, using MAFFT v.7 (Katoh and Standley, 2013). Gene trees were then generated from each alignment, using IQ‐TREE with automatic selection of the nucleotide substitution model and 1000 ultrafast bootstrap replicates (Kalyaanamoorthy et al., 2017; Minh et al., 2020).

## RESULTS

### A well‐resolved, multi‐individual reference phylogenomic framework

Illumina data obtained from herbarium leaf samples were generally of high quality, as the number of Angiosperms353 loci recovered ranged from 77 to 350 (out of 350), with a median of 334.5 and an average of 317 loci (Appendix S2). Nine samples could not be subjected to genome skimming, resulting in 0 to 23 plastid loci (out of 177) being recovered, while other samples had 21 to 177 plastid loci recovered, with a median of 177 and an average of 174 loci (Appendix S2). The ITS region could be retrieved from all samples with genome skimming data available, and from three out of the nine samples without such data available (Appendix S2).

**Figure 2:**
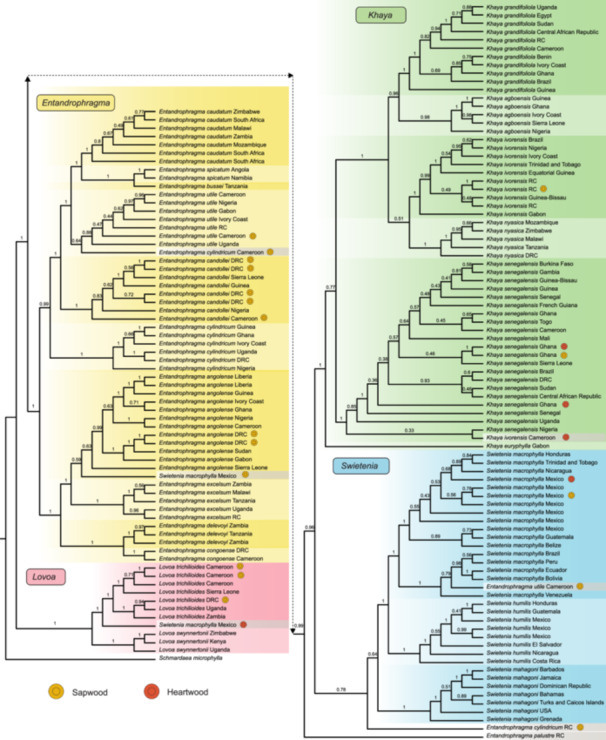
Intra‐ and interspecific relationships in four Meliaceae genera based on the paralog‐inclusive analysis of 343 nuclear genes. Numbers on branches indicate local posterior probabilities. Circles indicate sapwood (yellow) and heartwood (brown) samples. Colors and shades delimit genera and species, respectively, while gray highlights samples falling away from their clade.

Paralog‐inclusive (PI; Figure 2 and Appendix S4) and paralog‐exclusive (PE; Appendix S4) nuclear phylogenetic trees were highly similar for relationships between species and genera. The only difference was that *Khaya nyasica* Stapf ex Baker f. was sister to *K. ivorensis* in the PI tree but to *K. agboensis* A.Chev. and *K. grandifoliola* C.DC. in the PE tree, with weak support in both cases (i.e., local posterior probability [LPP] < 0.9). In the PI tree, all species and all genera but one were monophyletic when not considering the placement of wood samples (described in the next section), in most cases with strong support (LPP ≥ 0.9). The only exception was *Entandrophragma palustre* grouping with *Swietenia* and *Khaya* (LPP = 0.99), possibly because of low data recovery (Appendix S2). Species monophyly was lower in the PE tree (Appendix S4). In the plastome tree, all *Lovoa* and most *Entandrophragma* species were monophyletic but none of the *Khaya* or *Swietenia* species were (Appendix S4). In the ITS tree (Appendix S4), all *Lovoa* and all *Entandrophragma* species were monophyletic when not considering wood samples, while *K. ivorensis* and *S. mahagoni* (L.) Jacq. were the only monophyletic species of their respective genera.

### Wood identification using Hyb‐Seq data

The number of Angiosperms353 loci recovered from sapwood samples ranged from 0 to 349, with a median of 296 and an average of 213, while the number of recovered plastid loci ranged from 0 to 177, with a median of 54.5 and an average of 81 (Appendix S2). For heartwood samples, the number of Angiosperms353 loci recovered ranged from 0 to 296, with a median of 0 and an average of 52, while the number of recovered plastid loci ranged from 0 to 34, with a median of 0 and an average of five (Appendix S2). The ITS region was recovered for four out of 13 heartwood samples and 14 out of 20 sapwood samples (Appendix S2).

**Figure 3:**
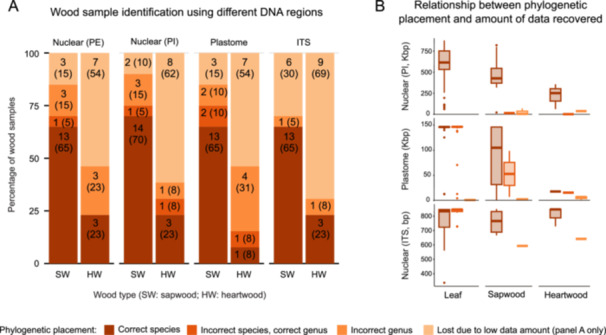
Identification of wood samples. (A) Placement accuracy of the wood samples depending on the genetic regions analyzed. The numbers outside brackets represent numbers of samples while the numbers inside brackets are the corresponding percentages. (B) Placement accuracy of all samples depending on data recovery. Recovery is measured as the cumulative length of the DNA regions recovered in kilo base pairs or base pairs. PE: paralog‐exclusive, PI: paralog‐inclusive. Placement categories indicate whether, based on its most closely related non‐wood (reference) sample, the sample was recovered in the right species, the right genus, neither, or if it was excluded from the phylogenetic tree due to insufficient data recovery.

Nuclear Angiosperms353 regions were only slightly better than ITS or the plastome at accurately placing wood samples in the phylogeny (Figure 3A). Regardless of the tissue, samples with extremely low recovery were consistently recovered outside of their species or even genus (Figure 3B). For the plastome and ITS regions, accuracy of identification was additionally genus dependent: some *Khaya* and *Swietenia* samples with good recovery did not group with the right species, whereas this was less often seen for *Entandrophragma* and never observed for *Lovoa* (Appendix S5). Sapwood samples were less frequently lost during data cleaning and were more frequently identified to species level than heartwood samples (Figure 3A), likely because the latter tended to have a worse region recovery (Figure 3B).

### DNA barcoding potential of reference loci

**Figure 4:**
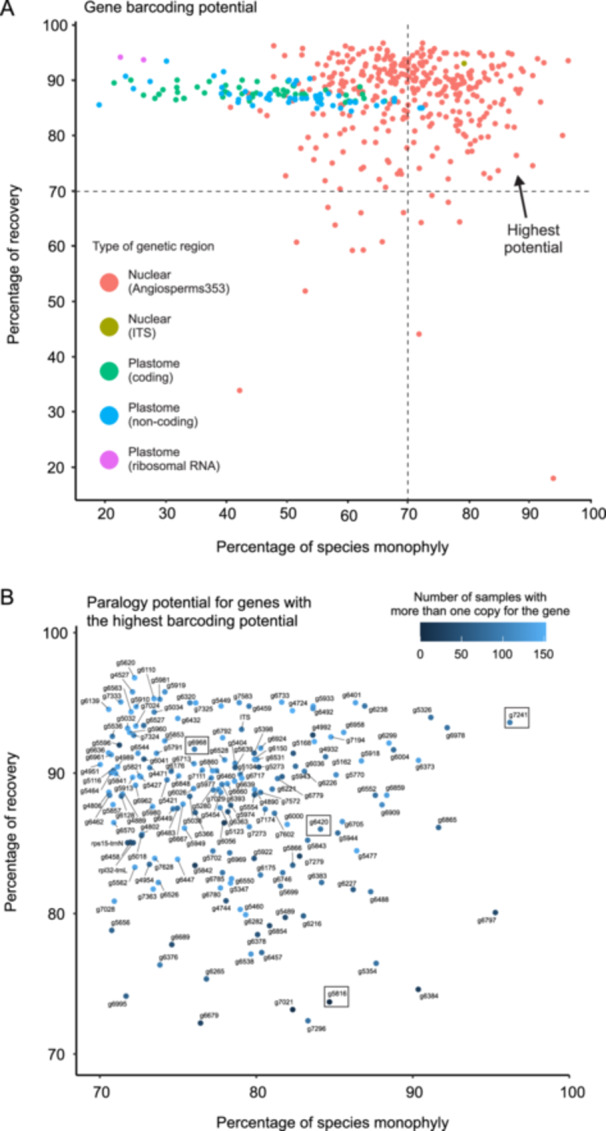
Potential of single DNA regions to serve as DNA barcodes in four Meliaceae genera. (A) Barcoding potential of all genes. (B) Genes with the highest barcoding potential (i.e., at least 70% of recovery and 70% of monophyly), colored by their paralogy potential. Boxes indicate the genes in which new DNA barcodes were designed for this study. The percentage of recovery was obtained by calculating the percentage of individuals of each species present in the gene tree and averaging this percentage across species. The percentage of species monophyly was obtained by calculating the percentage of individuals of each species present in the largest clade made by individuals of this species in the gene tree and averaging this percentage across species (see Methods).

We estimated the potential for DNA barcoding of each nuclear and plastid loci based on two criteria: (1) the rate of recovery of the locus among our samples, and (2) the rate of monophyly (see Methods and Discussion for details). Species were more often recovered as monophyletic with nuclear loci than with plastid loci (Figure 4A). Among the 174 loci with high barcoding potential (i.e., with rates superior to 70% for both criteria), only two were plastid while the rest were nuclear regions, including ITS (Figure 4A). The best gene according to both criteria was the Angiosperms353 gene “7241” (Figure 4B). This gene and the other genes with high barcoding potential all appeared potentially paralogous to some degree (i.e., CAPTUS assembled more than one sequence for the gene in at least one sample; Figure 4B). The only exception was the Angiosperms353 gene “5816,” which appeared to only be present in one copy in all samples, but had a relatively low barcoding potential (Figure 4B).

To evaluate how gene selection according to our criteria influenced barcode design and performance in the lab, we selected four genes for barcode design: gene 7241 (high barcoding and high paralogy potentials), gene 5816 (low barcoding and low paralogy potentials), and two other genes with intermediate barcoding and paralogy potentials, i.e., Angiosperms353 genes “6420” and “6968” (Figure 4B). The Gene Ontology (GO) terms of the four genes were obtained from the study describing the Angiosperms353 bait kit (Johnson et al., 2019). Gene 5816 (AT3G52640 in *Arabidopsis thaliana*) appears to code for an endoprotease known as NCT/Nicastrin (https://www.uniprot.org/uniprotkb/Q8GUM5/entry; https://amigo.geneontology.org/amigo/gene_product/AGI_LocusCode:AT3G52640; accessed on 17 January 2025). Gene 6420 (AT1G06240) appears to code for a protein of unknown function (see Extensive Annotation in Supplementary File 1 in Johnson et al., 2019). Gene 6968 (AT5G54290) appears to code for a thylakoid membrane protein known as CcdA (https://amigo.geneontology.org/amigo/gene_product/AGI_LocusCode:AT5G54290; accessed on 17 January 2025). Gene 7241 (AT4G01935) appears to code for a protein of unknown function (https://amigo.geneontology.org/amigo/gene_product/AGI_LocusCode:AT4G01935; accessed on 25 June 2025). Until further confirmation is provided, and for compatibility with other studies using Angiosperms353, we continue to call these genes 5816, 6420, 6968, and 7241.

### Candidate barcodes and primers

The sequence alignments of the four selected genes were used to design primers to amplify 12 candidate barcodes, i.e., short sections with high variation among species: 7241_r1, 7241_r2, 7241_r3, 5816_r1, 5816_r2, 5816_r4, 6420_r1, 6420_r2, 6968_r1, 6968_r2, 6968_r3a, and 6968_r3b (Appendix S6; see Methods for details). The primers and informativeness of the candidate barcodes across the focus genera are presented in Appendix S7. The same information is also provided for ITS1 and *trnL‐trnF* to allow comparison with these regions commonly used in plant phylogenetic or DNA barcoding studies. The candidate barcodes have median lengths of 164–397 bp, compared with 368–425 bp for ITS1 and *trnL‐trnF*. When looking at all the genera together, all candidate barcodes have more informative sites than *trnL‐trnF*, and two (6968_r2 and 6968_r3a) have more than ITS1 (139–145 vs. 136). These relative differences are maintained when looking at individual genera (Appendix S7).

Phylogenetic trees of our reference samples inferred from the candidate barcodes show variable degrees of resolution (Appendix S8). All barcode trees derived from genes 5816, 6420, and 7241 recover genera in separate clades, while the other trees show cases of genus polyphyly or paraphyly. Based on our reference samples, the candidate barcodes providing the highest average percentage of monophyly across all species are 6420_r2, 5816_r2, 7241_r3, and 7241_r2, with 80–87% (Appendix S7). Barcodes *trnL‐trnF*, 6868_r3a, and 6968_r3b do not provide good interspecific resolution (<60% average monophyly), even when combining 6868_r3a and 6968_r3b (Appendix S8). The best barcode at resolving *Entandrophragma* species is 5816_r2 (with 100% monophyly on average), and most other barcodes perform well in this genus (>70% average monophyly) except 6868_r3a, 6868_r3b, 6868_r3a and 6868_r3b combined, and *trnL‐trnF* (Appendix S7). Barcodes 7241_r1 and 7241_r3 are best at distinguishing *Swietenia* species (>90% average monophyly), although *S. humilis* Zucc. remains paraphyletic in all but two barcode trees (7241_r2 and 7241_r3). Both *Lovoa* species are recovered as monophyletic with all barcodes except 5816_r4. None of the candidate barcodes can fully resolve *Khaya* species in distinct clades (Appendix S8), with only 7241_r2 reaching more than 70% average monophyly in this genus (Appendix S7).

### Wood identification using the candidate barcodes

**Figure 5:**
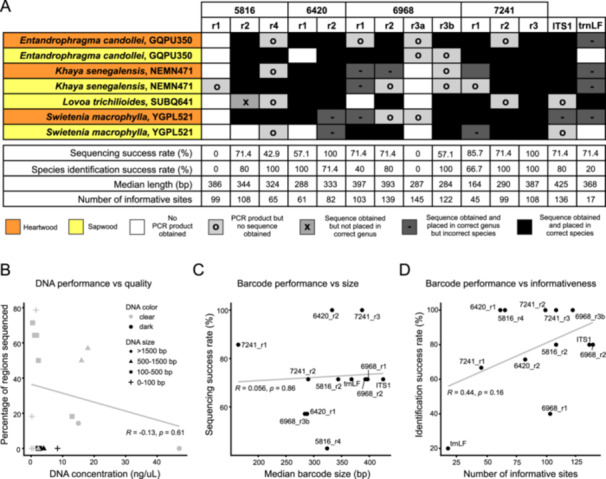
Identification of wood samples using PCR and Sanger sequencing of selected DNA regions. (A) Summary of the success of the PCR, sequencing, and phylogenetic analysis of the 12 new candidate barcodes, the ITS1 region, and the *trnL‐trnF* region for four species of the focus genera. The heatmap depicts the most advanced test stage successfully achieved with at least one sample of the species for each wood type available (HW: heartwood, SW: sapwood), for each region. “No PCR product” means that PCR failed; “PCR product” means that sequencing failed; and “Sequence,” “Correct genus,” and “Correct species” mean, respectively, that a sequence was produced but did not fall in the correct genus in the phylogenetic tree, that it fell in the right genus but not the right species, and that it fell in the right species. The heatmap shows results across all DNA extracts tested, which means that some regions or species were tested more than others. However, the sequencing and identification success rates provided have been calculated by using only the results from the seven best‐performing DNAs that represented the seven species/wood combinations, so that rates are comparable between regions. Median lengths are the median lengths of the region in the reference alignments created from the target capture sequencing data, and the number of informative sites was also calculated from these reference alignments. (B) Relationship between DNA quality and percentage of regions successfully sequenced among those tested (see Appendix S3 for details). (C) Relationship between barcode size and sequencing success rate as defined in A. (D) Relationship between barcode informativeness and identification success rate as defined in A. Barcodes 5816_r1 and 6968_r3a, which had outlier sequencing and identification success rates of 0%, were omitted from the plots and tests presented on panels C and D.

PCR and Sanger sequencing of at least one region among the 12 candidate barcodes, ITS1, and *trnL‐trnF* was successful in four out of the nine heartwood and in six out of the 10 sapwood samples tested, representing four out of the six species tested (Appendix S3). The species that failed all tests were *Entandrophragma cylindricum* and *Khaya ivorensis*. Between two and 11 candidate barcodes could be successfully sequenced from heartwood samples, and between one and nine could be sequenced from sapwood samples (Figure 5A, Appendix S3). Many samples from which no barcode could be sequenced had low‐concentrated, highly fragmented DNA (Figure 5B). However, many low‐concentrated DNA extracts had high sequencing success (including some containing mostly very short fragments), and having highly concentrated DNA with larger fragments was not a guarantee of success (Figure 5B; Appendices S3, S9, S10). No barcode could be amplified from dark brown or black DNA extracts (Appendices S3, S10), but clear DNA was not a guarantee of success. There was no strong correlation between sequencing success and DNA purity assessed from the ratios of the absorbance of DNA extracts at 260 nm and of their absorbances at 280 nm and 230 nm (Appendices S3, S10).

To explore whether the candidate barcodes had different sequencing and identification success rates regardless of DNA quality, we compared their performance on the seven DNA extracts that had yielded good sequences for at least half of the regions. This included DNA from sapwood and heartwood of *Entandrophragma candollei*, *Khaya senegalensis*, and *Swietenia macrophylla* and from sapwood of *Lovoa trichilioides* (Figure 5A, Appendix S3). Barcode performance on these samples was evaluated based on sequencing success rate (percentage of DNA extracts that yielded a sequence for the barcode; Appendix S3) and identification success rate (percentage of DNA sequences that grouped with the correct species in the barcode phylogenetic tree; Appendix S11). There was no strong correlation between barcode size and sequencing success rate (Figure 5C), nor between barcode informativeness and identification success rate (Figure 5D). Only one region (7241_r3) had a rate of 100% successful PCR amplification and sequencing, coupled with 100% accurate phylogenetic placement at the species level, while two other highly performing regions had 100% sequencing success and 71.4% identification success (6420_r2) or vice versa (7241_r2; Figure 5A). Three regions showed intermediate performance in both aspects (5816_r2, 6968_r2, ITS1), with success rates of 71% and 80% for sequencing and species‐level identification, respectively (failures were not associated with a specific sample or species; Figure 5A). These results were consistent with the high resolution of the reference phylogenetic trees produced from these regions (Appendix S8). The phylogenetic tree obtained by combining the alignments of the three candidate barcodes that performed best on our test samples offered high species‐level resolution, with 15 out of 20 species represented by multiple individuals being monophyletic, the exceptions being *E. angolense* C.DC., *K*. cf. *anthotheca* (each with two “rogue” individuals placing away from the main species group), *K. grandifoliola*, *K. agboensis*, and *K. nyasica* (each with one rogue individual; Appendices S7, S12). No further resolution was gained when building a tree from the concatenated alignments of the three best candidate barcodes in combination with the three candidate barcodes with intermediate performance (Appendix S12). Three regions (6968_r3b, 5816_r4, 6420_r1) had low sequencing success (43–57%), and although they had maximal identification success rates on the sequences that were obtained (Figure 5A), the reference phylogenies for these regions were poorly resolved (Appendix S8). Three regions (6968_r1, 7241_r1, *trnL‐trnF*) had relatively high sequencing success rates (71–86%) but showed low identification success rates (20–67%) on our test species, which was also expected from the low resolution of their reference trees (Figure 5A, Appendix S8). Finally, two regions did not work well with the current protocols (5816_r1, 6968_r3a), with no exploitable sequences being generated (Figure 5A, Appendix S3).

## DISCUSSION

### Using Hyb‐Seq to build DNA barcoding reference frameworks

Our approach to using Hyb‐Seq for the development of DNA barcoding resources starts with inferring a well‐resolved evolutionary framework of the focus species, their look‐alikes, and their close relatives. Generating such a framework from vouchered individuals across species distribution ranges increases its reliability and potential applicability for DNA barcoding (Collins and Cruickshank, 2013; Coissac et al., 2016) while providing higher resolution in our understanding of the evolutionary history of genes and species in the group of interest. Beyond supporting the selection of new DNA barcodes (see below), this can facilitate the interpretation of DNA barcoding results by providing new light on species relationships, unresolved taxonomic questions, and possible cases of paralogy and reticulation.

Phylogenies based on our Hyb‐Seq data confirm results from previous studies (Muellner et al., 2003, 2011; Monthe et al., 2019) and clarify species‐level relationships in *Swietenia*, *Entandrophragma*, and *Khaya* that were previously unresolved. Despite the increase in phylogenetic resolution obtained from the analysis of hundreds of genes, the precise placements of *E. palustre*, *K. nyasica*, and *K. euryphylla* remain unclear, and the relationship of *K. anthotheca* to the other species could not be inferred at all given the uncertain identity of the specimens we had sampled to represent this species (Appendix S1). These remaining uncertainties align with previous studies showing that *E. palustre* is a poorly understood species and that *Khaya* species likely originated recently and may have undergone reticulation events (Monthe et al., 2019; Bouka et al., 2022). Although sequencing other genomic regions may help further clarify the phylogenetic placements of these species, the complexity of their taxonomy (Monthe et al., 2019; Bouka et al., 2022) suggests that the biggest improvements in our understanding may be achieved through sequencing more accessions that better represent their genetic, geographic, and morphological diversity. The impacts of these sampling gaps on barcode selection and species identification are discussed below.

### From reference loci to candidate barcodes and primers

The generation of a large reference DNA dataset enabled the search for individual DNA barcodes suitable for PCR amplification and Sanger sequencing from fragmented DNA. The criteria we used to characterize the barcoding potential of each locus are based on two practical considerations: the availability of reference samples to inform barcode delimitation and primer design (recovery criterion), and the performance of the locus at distinguishing species from each other (monophyly criterion). Both criteria aim at reducing the number of barcodes to be tested in the lab by highlighting genes that are likely to contain subregions that might be suitable barcodes. However, these criteria cannot function as direct predictors of barcoding success. The recovery criterion is used so that the barcoding potential of genes can be assessed based on as many accessions as possible, but recovery of the gene through Hyb‐Seq does not provide any indication of the ease with which the candidate barcodes designed in this gene can be retrieved from test samples using PCR and Sanger sequencing. The monophyly rate of a gene serves as a good indication of its information content, but depending on the location and nature (homologous or not) of the informative sites, genes with a high degree of monophyly may not necessarily yield barcodes with high discrimination power, and genes with low monophyly rates may contain subregions with high amounts of phylogenetic information. Moreover, the rate of monophyly can be misleading if the sampling is insufficient to capture inter‐ and intraspecific variation or if some species are non‐monophyletic (see below). Despite these limitations, the gene that had the highest degree of monophyly and among the highest percentages of recovery (Figure 4) yielded two of the three candidate barcodes that performed best on our dataset (7241_r2 and 7241_r3), empirically validating our approach to gene and barcode selection.

Beyond potential conceptual limitations, an issue likely to be encountered to some degree by any user given the unequal availability of taxa in collections is the presence of gaps in the sampling of reference specimens. Here, four species could only be sampled once (*E. bussei*, *E. palustre*, *K. euryphylla*) or not at all (*K. madagascariensis*), and expectations about the monophyly of specimens tentatively assigned to *K. anthotheca* were unclear. The monophyly criterion could still be applied to most species with confidence, and our gene selection included not only the best gene but other genes with different properties as a test case, so it is likely that the selection we made here would be robust to the addition of samples from under‐represented species. However, it cannot be ruled out that adding more samples from these species could yield different estimates of gene monophyly percentage and barcoding potential and therefore lead to the design of a different barcode set, highlighting the importance of building a reference dataset that is as well understood and comprehensive as possible.

In our workflow, paralogy checks are used to inform the choice of candidates among the best genes. We found that for our test genera the amount of potential paralogy detected (Figure 4B) was not a predictor of PCR or Sanger sequencing failure (Figure 5A). This may be because the different gene copies assembled resulted from phenomena other than paralogy (e.g., mutations, low levels of inter‐sample contamination, or sequencing errors) and/or because our primers were designed on the alignments including only orthologous copies. To completely avoid the risk of Sanger sequencing failure due to paralogy, our workflow could be expanded so that the paralogy flags obtained from the Hyb‐Seq data analysis pipeline would be used as a third criterion to rule out genes with more than one copy. However, this may result in excluding highly performing barcodes. Alternatively, copy‐specific primers could be designed based on the reference sequences available for the different copies. Importantly, Sanger sequencing validation confirms amplification and sequence recovery but does not necessarily resolve underlying paralogy, as dominant copies may be preferentially amplified, leaving paralogous variation undetected.

Another criterion that is decisive for timber DNA barcoding is the length of the barcodes (Jiao et al., 2020), especially if these are to be obtained from degraded DNA by PCR and Sanger sequencing, which is often desirable in contexts where resources to perform high‐throughput short‐read sequencing are limited (Tonouéwa et al., 2024). The regions recovered with the Angiosperms353 and many other target capture sequencing kits are typically longer than 500 bp (Johnson et al., 2019), especially when regions flanking the targets are also recovered, which was true in our case. Here, we circumvented this limitation by selecting four loci of high barcoding potential and manually searching them for shorter (150–400 bp) regions (candidate barcodes) with species‐specific variation surrounded by more conserved regions for primer design. To further streamline the search for barcodes, a future iteration of this workflow could implement an automatic screening of high‐potential loci to identify informative regions of a user‐defined size.

### Barcode performance in silico and validation on wood DNA

The bioinformatic workflow presented here enabled the identification of loci that recover monophyletic groupings for the sampled taxa, both individually and in combination (Appendices S7, S8, S12). However, such monophyly should be interpreted as a proxy metric that is contingent on taxon sampling, locus selection, and phylogenetic resolution. These loci therefore represent promising candidate barcodes within the current dataset, rather than definitive indicators of species‐level discrimination. Although monophyly‐based identification can theoretically be performed with a phylogeny comprising a single reference individual per species, this approach can fail if interspecific genetic variation is low relative to intraspecific variation (small barcoding gap), especially if the latter is not captured in the reference dataset (Meyer and Paulay, 2005). Consequently, the estimates of barcode performance and discriminatory power presented here are conditional on the current sampling and may change with expanded representation of intraspecific diversity. The performance of our candidate barcodes at identifying samples of species currently represented by a single sample in our reference dataset (i.e., *E. bussei*, *E. palustre*, and *K. euryphylla*) remains unclear, and samples from *K. anthotheca* and *K. madagascariensis* cannot currently be identified because confidently identified specimens from these species are missing from the reference dataset. More crucially, the absence of these two species from the reference means that an assignment of any unknown *Khaya* sample to these species cannot be completely ruled out. This limitation highlights that barcode‐based identification frameworks relying on phylogenetic placement are inherently sensitive to incomplete taxon representation and may yield ambiguous or incorrect assignments when reference coverage is incomplete. These issues do not undermine the general approach to developing DNA barcoding resources presented here but underscore that unambiguous sample identification can only be performed in a context where species delimitations are well resolved and intra‐ and interspecific genetic variation are well represented in the reference dataset. In the case of our focus genera, more reference data should ideally be generated to better represent the species mentioned above, and possibly also to capture even more genetic diversity from species with wide ranges (e.g., *Entandrophragma angolense*, *E. candollei*, *E. cylindricum*, and *L. trichilioides*). To enable robust identifications, however, this sampling will have to be informed by further taxonomic work on the poorly understood species. The lack of consistent monophyly in *Khaya* also suggests limitations in resolving species boundaries using the selected loci and highlights potential challenges associated with incomplete lineage sorting, introgression, or insufficient phylogenetic signal that may only be overcome by designing other barcodes in addition to sampling more individuals.

To test if the new candidate barcodes could potentially be deployed to audit timber supply chains where species from different focus genera are intermingled, further validation in the lab should ideally be performed on all species from the reference. This is required to ensure that the selected barcodes can be amplified from the wood of all relevant species using PCR and to tweak PCR protocols or primer sequences as needed, for instance, if some species have mutations in the primer regions. As a first step in this direction, our test wood samples were selected to represent the diversity of DNA qualities and the taxonomic breadth of the species that may be found in timber supply chains including the focus genera (Lowe et al., 2016). Our bioinformatic analyses (Figure 4) and tests on wood DNA (Figure 5) confirm that isolated plastid regions are usually not informative enough and that, while ITS1 remains an informative standard barcode for the focus genera, it can be superseded by our best candidate barcodes both in terms of sequencing and identification success rates on wood. Our results also indicate that 6420_r2, 7241_r2, and 7241_r3 may be suitable for the identification of wood samples of the focus genera, given their high species‐level identification and sequencing success rates on the test wood DNA. However, these candidate barcodes could only be lab‐tested on six species (*Entandrophragma cylindricum*, *E. candollei*, *Khaya ivorensis*, *K. senegalensis*, *Lovoa trichilioides*, and *Swietenia macrophylla*), so further studies will be needed to explore broader applicability across the family. Additional trials on more sample types and further validation across broader taxonomic and geographic sampling will also be essential to more thoroughly evaluate whether PCR and Sanger sequencing of these candidate barcodes could be applied to supply chain monitoring in the future, as species and products to be identified vary depending on the country and users (Momballa‐Mbun et al., 2023; Tonouéwa et al., 2024).

By allowing the identification and testing of multiple candidate DNA barcodes in a single framework, our workflow simplifies investigating the drivers of poor barcode performance and identifying the most promising areas for protocol optimization. Crucially, we show that candidate barcodes with high species‐level resolution in reference trees may not easily be sequenced in practice (Figure 5). DNA fragmentation and impurity are often responsible for the failure of PCR‐based wood DNA barcoding (Jiao et al., 2020). Barcode length did not seem to be a limiting factor in our case; this was likely because although most of our wood DNA samples were degraded, they often contained at least some fragments longer than the barcodes. However, further tests on a larger number of wood samples may reveal the need for designing intermediate primers so that the candidate barcodes identified here could be amplified in two steps from samples with even more degraded DNA. Our assessment of DNA purity was only based on absorbance ratios and color, with only the latter being potentially associated with sequencing failure. Future studies will be required to understand the nature of this association, which might ultimately enable the development of tailored DNA cleaning protocols and an increase in sequencing success rates on wood samples.

### A general workflow to unlock DNA barcoding of timber and beyond

This study demonstrates how Hyb‐Seq data may be utilized to develop cost‐efficient DNA barcoding through the generation of multi‐species, multi‐individual reference datasets and the identification and test of DNA barcodes (Figure 1). This set of candidate barcodes and associated reference data and lab protocols (Appendix S1) constitute a previously unavailable, unified go‐to DNA barcoding resource for the focus genera, on which future studies including more reference and wood test samples can build. By constructing our reference dataset entirely from herbarium specimens, we illustrate the crucial role that well‐curated and well‐studied museum collections can play in supporting the development of DNA barcoding resources and potential downstream applications. The gaps remaining in our reference may have consequences for barcode selection and identification accuracy. Indeed, uneven and limited sampling across taxa may influence locus ranking and perceived barcode performance, potentially biasing the identification of high‐performing loci under the current dataset and highlighting the importance of having as comprehensive and representative a reference as possible. The advent of integrative taxonomy combining morphological and DNA surveys and its application to our focus genera, particularly *Khaya*, as initiated by Bouka et al. (2022), will be key to addressing shortcomings in our reference data. Such taxonomic work may in turn benefit from the well‐resolved phylogenies inferred in our study as a starting framework in which to place and understand unclear specimens, in a process of reciprocal illumination.

The workflow presented here could be repeated to develop DNA reference datasets and barcodes for other heavily exploited species. Given the wide adoption of the Angiosperms353 bait kit (Baker et al., 2022; Albreht et al., 2025 for *Pterocarpus*) and other kits specific to plant families containing highly valued species in need of trade monitoring (e.g., Crameri et al., 2022 for *Dalbergia* and other Fabaceae; Eserman et al., 2021 for orchids), there is already a profusion of genomic data available in public databases that could be exploited towards this goal. This would help make the most of the wealth of target capture sequencing and/or genome skimming data associated with vouchered specimens that have been published over the past decade for phylogenomic and population genomic studies. In turn, it may support the deployment of DNA barcoding in contexts where its application is theoretically desirable but has been difficult in practice, such as food authentication, the traceability of plant‐based manufactured products, or the control of ornamental plant poaching (Kress, 2017).

## AUTHOR CONTRIBUTIONS

S.B. and V.D. conceived the research, acquired the funding, and managed the project with input from D.N.B. and J.L.; C.Q.‐B., L.C., S.B., J.L., and B.M. performed the experiments; B.A.I., G.U.D.B., E.E., C.G.M., M.R., P.Y., and G.H.Z.T. provided samples; data analysis was mainly done by S.B., with contributions from J.L., L.C., and C.Q.‐B.; S.B. and V.D. wrote the first draft of the manuscript with input from D.N.B. and J.L.; L.C., P.G., G.U.D.B., and M.R. provided feedback on the manuscript. All authors approved the final version of the manuscript.

## Supporting information


**Appendix S1:** Supplementary methods including wood DNA extraction protocol.

**Appendix S2:** Samples used to create the DNA reference dataset.

**Appendix S3:** Wood DNA extractions performed in this study with PCR, sequencing, and species assignment results for 19 DNA extracts.

**Appendix S4:** Intra‐ and interspecific relationships in four Meliaceae genera based on the paralog‐inclusive and paralog‐exclusive analyses of 343 and 350 nuclear genes, respectively, the analysis of 177 plastome regions, and the analysis of the ITS region.

**Appendix S5:** Phylogenetic placement accuracy depending on region recovery and genus.

**Appendix S6:** Schematic representation of the 12 new barcodes and corresponding primers.

**Appendix S7:** Characteristics of the 12 new and two traditional DNA barcodes.

**Appendix S8:** Phylogenetic resolution obtained using the new and traditional barcodes.

**Appendix S9:** Size of the DNA used for PCR and Sanger sequencing of the barcodes.

**Appendix S10:** Relationship between DNA quality and PCR and Sanger sequencing results.

**Appendix S11:** Placement of wood samples using the 12 new DNA barcodes, ITS1, and the *trnL‐trnF* region.

**Appendix S12:** Phylogenetic resolution obtained when combining the best three or six barcodes.

## Data Availability

Raw Illumina sequence reads are deposited in the National Center for Biotechnology Information Sequence Read Archive (NCBI SRA; BioProject PRJNA1185931). SRA accession numbers and related metadata including voucher information can be found in Appendix 1 for herbarium specimens and Appendix S2 for wood samples. For Sanger sequences obtained from wood samples, GenBank accession numbers and related metadata including voucher information can be found in Appendix S3. Raw and final Sanger sequences, as well as all alignments and phylogenetic trees produced in this study are available at https://github.com/sidonieB/Bellot_al_Meliaceae_DNA_barcoding.

## Appendix 1 Herbarium specimens used in this study.^a^

**Table:**

Species	Voucher	Country	SRA accession numbers (Angiosperms353/skimming)
*Entandrophragma angolense*	Etuge, M. 6637 (K)	Cameroon	SRR31348580/SRR31348663
*Entandrophragma angolense*	A. J. M. Leeuwenberg 2493 (K)	Cote d'Ivoire	SRR31348568/SRR31348534
*Entandrophragma angolense*	F. J. Breteler 15422 (K)	Gabon	SRR31348449/SRR31348441
*Entandrophragma angolense*	A. E. Kitson 1230 (K)	Ghana	SRR31348557/SRR31348533
*Entandrophragma angolense*	Burgt, X. M. van der 2286 (K)	Guinea	SRR31348582/SRR31348536
*Entandrophragma angolense*	Elisha, E. 1035555 (K)	Nigeria	SRR31348397/SRR31348418
*Entandrophragma angolense*	D. Small 715 (K)	Sierra Leone	SRR31348462/SRR31348358
*Entandrophragma angolense*	T. F. Chipp 13 (K)	Sudan	SRR31348365/SRR31348436
*Entandrophragma bussei*	S. Bidgood et al. 1156 (K)	Tanzania	SRR31348525/SRR31348417
*Entandrophragma candollei*	Haba, P. M. 727 (K)	Guinea	SRR31348514/SRR31348416
*Entandrophragma candollei*	J. P. M. Brenan et al. 8438 (K)	Nigeria	SRR31348528/SRR31348370
*Entandrophragma candollei*	X. M. van der Burgt 1649 (K)	Sierra Leone	SRR31348581/SRR31348548
*Entandrophragma caudatum*	P. Thopham 741 (K)	Malawi	SRR31348604/SRR31348405
*Entandrophragma caudatum*	B. T. Styles 3784 (K)	Mozambique	SRR31348607/SRR31348409
*Entandrophragma caudatum*	J. Vahrmeyer + Joynt 177 (K)	South Africa	SRR31348527/SRR31348530
*Entandrophragma caudatum*	F. White 10488 (K)	South Africa	SRR31348610/SRR31348411
*Entandrophragma caudatum*	Mabatha F.W., Nkuna L.A., van Slageren M. 2272 (K)	South Africa	SRR31348608/SRR31348410
*Entandrophragma caudatum*	F. White 1977 (K)	Zambia	SRR31348606/SRR31348407
*Entandrophragma caudatum*	R. B. Drummond & R. O. B. Rutherford‐Smith 7543 (K)	Zimbabwe	SRR31348605/SRR31348406
*Entandrophragma congoense*	R. LETOUZEY 14499 (K)	Cameroon	SRR31348615/SRR31348413
*Entandrophragma congoense*	R. Dechamps 164 (K)	Democratic Republic of the Congo	SRR31348488/SRR31348412
*Entandrophragma cylindricum*	A. J. M. Leeuwenberg 2483 (K)	Cote d'Ivoire	SRR31348658/SRR31348361
*Entandrophragma cylindricum*	Terese B. Hart 417 (K)	Democratic Republic of the Congo	SRR31348599/SRR31348404
*Entandrophragma cylindricum*	B. A. Krukoff 40 (K)	Ghana	SRR31348647/SRR31348539
*Entandrophragma cylindricum*	Haba, P. M. 728 (K)	Guinea	SRR31348382/SRR31348542
*Entandrophragma cylindricum*	B. O. Darmola 457194 (K)	Nigeria	SRR31348593/SRR31348666
*Entandrophragma cylindricum*	B. T. Styles 52 (K)	Uganda	SRR31348598/NA
*Entandrophragma delevoyi*	A. A. Bullock 2071 (K)	Tanzania	SRR31348597/SRR31348403
*Entandrophragma delevoyi*	C.E. Duff 150/33 (K)	Zambia	SRR31348596/SRR31348402
*Entandrophragma delevoyi*	W. L. Astle 865 (K)	Zambia	SRR31348595/SRR31348401
*Entandrophragma excelsum*	T. Muller 1608 (K)	Malawi	SRR31348384/SRR31348396
*Entandrophragma excelsum*	Herbier M. Reynders 208 (K)	Republic of the Congo	SRR31348387/SRR31348400
*Entandrophragma excelsum*	J. M. Grimshaw 9373 (K)	Tanzania	SRR31348385/SRR31348398
*Entandrophragma excelsum*	B. T. Styles 319 (K)	Uganda	SRR31348386/SRR31348399
*Entandrophragma excelsum*	D.B.F. F5126 (K)	Zambia	SRR31348383/SRR31348395
*Entandrophragma palustre*	Germain 8395 (K)	Republic of the Congo	SRR31348381/NA
*Entandrophragma spicatum*	F. Crawford 456 (K)	Angola	SRR31348380/SRR31348394
*Entandrophragma spicatum*	D.A.H. Taylor 296 (K)	Namibia	SRR31348379/SRR31348393
*Entandrophragma utile*	F. J. Breteler 2165 (K)	Cameroon	SRR31348377/SRR31348392
*Entandrophragma utile*	A. J. M. Leeuwenberg 2510 (K)	Cote d'Ivoire	SRR31348477/SRR31348545
*Entandrophragma utile*	B. A. Krukoff 139 (K)	Gabon	SRR31348376/SRR31348391
*Entandrophragma utile*	M. G. Latilo 32980 (K)	Nigeria	SRR31348600/SRR31348364
*Entandrophragma utile*	J. Wagemans 1497 (K)	Republic of the Congo	SRR31348375/SRR31348390
*Entandrophragma utile*	B. T. Styles 109 (K)	Uganda	SRR31348374/SRR31348389
*Khaya cf. anthotheca*	A. Leonard 3242 (K)	Democratic Republic of the Congo	SRR31348650/SRR31348519
*Khaya cf. anthotheca*	S.R. Semsei 51076 (K)	Kenya	SRR31348657/SRR31348526
*Khaya cf. anthotheca*	B.T. Styles 261 (K)	Tanzania	SRR31348656/SRR31348524
*Khaya cf. anthotheca*	B.T. Styles 125 (K)	Uganda	SRR31348372/SRR31348388
*Khaya agboensis*	J.J.F.E. de Wilde 3744 (K)	Cote d'Ivoire	SRR31348653/SRR31348521
*Khaya agboensis*	G.W.A. 585 (K)	Ghana	SRR31348654/SRR31348522
*Khaya agboensis*	Haba P. M. 892 (K)	Guinea	SRR31348651/SRR31348520
*Khaya agboensis*	J.O. Amachi 38275 (K)	Nigeria	SRR31348652/NA
*Khaya agboensis*	J.S. Sawyerr F. H. K. 13601 (K)	Sierra Leone	SRR31348655/SRR31348523
*Khaya euryphylla*	Thomson 1 (K)	Gabon	SRR31348649/NA
*Khaya grandifoliola*	H. Ern 3172 (K)	Benin	SRR31348464/SRR31348510
*Khaya grandifoliola*	D.A. Folli 6785 (K)	Brazil	SRR31348643/SRR31348516
*Khaya grandifoliola*	M.G. Latilo & B.O. Daramol 34481 (K)	Cameroon	SRR31348648/SRR31348518
*Khaya grandifoliola*	John M. Fay 4164 (K)	Central African Republic	SRR31348463/SRR31348509
*Khaya grandifoliola*	Aubreville 63 (K)	Cote d'Ivoire	SRR31348466/SRR31348512
*Khaya grandifoliola*	Min of Agrenltine n/a (K)	Egypt	SRR31348468/SRR31348515
*Khaya grandifoliola*	G. Vigne 1803 (K)	Ghana	SRR31348467/SRR31348513
*Khaya grandifoliola*	Aug. Chevalier 20687 (K)	Guinea	SRR31348465/SRR31348511
*Khaya grandifoliola*	R. Germain 4166 (K)	Republic of the Congo	SRR31348646/NA
*Khaya grandifoliola*	L. Turner 184 (K)	Sudan	SRR31348645/SRR31348517
*Khaya grandifoliola*	B.T. Styles 263 (K)	Uganda	SRR31348589/SRR31348642
*Khaya ivorensis*	G.S. Siqueira & G. Terra 14357 (K)	Brazil	SRR31348457/SRR31348505
*Khaya ivorensis*	B. A. Krukoff 70 (K)	Cote d'Ivoire	SRR31348455/SRR31348502
*Khaya ivorensis*	M.F. Carvalho 2829 (K)	Equatorial Guinea	SRR31348594/SRR31348500
*Khaya ivorensis*	B.A. Krufoff 159 (K)	Gabon	SRR31348460/NA
*Khaya ivorensis*	M.T. Dawe 237 (K)	Guinea‐Bissau	SRR31348456/SRR31348504
*Khaya ivorensis*	J.P.M. Brenan, C.F.A. Onochie, E.W. Jones & P.W. Richards s.n. (K)	Nigeria	SRR31348453/SRR31348501
*Khaya ivorensis*	L. Toussaint 680 (K)	Republic of the Congo	SRR31348459/SRR31348507
*Khaya ivorensis*	F.C. Butlin 11972 (K)	Trinidad and Tobago	SRR31348458/SRR31348506
*Khaya nyasica*	J.J. Symoens 8803 (K)	Democratic Republic of the Congo	SRR31348592/SRR31348499
*Khaya nyasica*	J.D. & E.G. Chapman 8050 (K)	Malawi	SRR31348587/SRR31348640
*Khaya nyasica*	A. Gomes e Sousa 4326 (K)	Mozambique	SRR31348588/SRR31348641
*Khaya nyasica*	David A.H. Taylor 257 (K)	Tanzania	SRR31348591/SRR31348498
*Khaya nyasica*	D. F. Lovemore 327 (K)	Zimbabwe	SRR31348586/SRR31348639
*Khaya senegalensis*	G.S. Siqueira & G. Terra 14358 (K)	Brazil	SRR31348576/SRR31348632
*Khaya senegalensis*	A.J.M. Leeuwenberg 4318 (K)	Burkina Faso	SRR31348563/SRR31348621
*Khaya senegalensis*	M.G. Latilo & B.O. Daramola 34438 (K)	Cameroon	SRR31348584/SRR31348638
*Khaya senegalensis*	J. Michael Fay & Joel Doka 5269 (K)	Central African Republic	SRR31348579/SRR31348635
*Khaya senegalensis*	Mil 9717 (K)	Democratic Republic of the Congo	SRR31348583/SRR31348636
*Khaya senegalensis*	Oldeman 1258 (K)	French Guiana	SRR31348577/SRR31348633
*Khaya senegalensis*	J.P.Ruxton 162 (K)	Gambia	SRR31348571/SRR31348628
*Khaya senegalensis*	S. Kitson 702 (K)	Ghana	SRR31348574/SRR31348630
*Khaya senegalensis*	J.M. Daziel n/a (K)	Guinea	SRR31348566/SRR31348624
*Khaya senegalensis*	E. Santo 1946 (K)	Guinea‐Bissau	SRR31348565/SRR31348623
*Khaya senegalensis*	Bamps 2478 (K)	Mali	SRR31348570/SRR31348627
*Khaya senegalensis*	J.Lowe 2546 (K)	Nigeria	SRR31348573/NA
*Khaya senegalensis*	s.coll. s.n. 12/04/1951 (K)	Senegal	SRR31348575/SRR31348631
*Khaya senegalensis*	J. G. Adam 17935 (K)	Senegal	SRR31348572/SRR31348629
*Khaya senegalensis*	F.C. Deighton 5894 (K)	Sierra Leone	SRR31348564/SRR31348622
*Khaya senegalensis*	L. Turner 184 (K)	Sudan	SRR31348578/SRR31348634
*Khaya senegalensis*	B.T. Styles 2085 (K)	Togo	SRR31348567/SRR31348625
*Khaya senegalensis*	B.T. Styles 255 (K)	Uganda	SRR31348644/NA
*Lovoa swynnertonii*	Nzano 14716 (K)	Kenya	SRR31348561/SRR31348619
*Lovoa swynnertonii*	B.T. Styles 245a (K)	Uganda	SRR31348562/SRR31348620
*Lovoa swynnertonii*	H. Wild 2228 (K)	Zimbabwe	SRR31348560/SRR31348618
*Lovoa trichilioides*	M. Cheek 11533 (K)	Cameroon	SRR31348554/SRR31348616
*Lovoa trichilioides*	Luke, W.R.Q. 15234 (K)	Sierra Leone	SRR31348558/SRR31348617
*Lovoa trichilioides*	K. A. Lye 6240 (K)	Uganda	SRR31348552/SRR31348497
*Lovoa trichilioides*	J. Timberlake, M. Bingham & A. Cunningham 5825 (K)	Zambia	SRR31348553/SRR31348613
*Swietenia humilis*	D.H. Boshier 21 (K)	Costa Rica	SRR31348446/SRR31348489
*Swietenia humilis*	E. Mayorga 3869 (K)	El Salvador	SRR31348450/SRR31348492
*Swietenia humilis*	J.J. Castillo M. 1987 (K)	Guatemala	SRR31348448/SRR31348491
*Swietenia humilis*	D.H. Boshier 49 (K)	Honduras	SRR31348451/SRR31348493
*Swietenia humilis*	T.D. Pennington & J. Sarukhan K. 9219 (K)	Mexico	SRR31348550/SRR31348495
*Swietenia humilis*	B.T. Styles 113 (K)	Mexico	SRR31348551/SRR31348496
*Swietenia humilis*	T.D. Pennington & J. Sarukhan 9456 (K)	Mexico	SRR31348452/SRR31348494
*Swietenia humilis*	P. P. Moreno 23530 (K)	Nicaragua	SRR31348447/SRR31348490
*Swietenia macrophylla*	T. Sarkinen 804 (K)	Belize	SRR31348439/SRR31348483
*Swietenia macrophylla*	Israel G., Vargas C. & Claudia Jordan 6278 (K)	Bolivia	SRR31348426/SRR31348472
*Swietenia macrophylla*	B. Dubs 1719 (K)	Brazil	SRR31348431/SRR31348478
*Swietenia macrophylla*	J. Zuleta 9 (K)	Ecuador	SRR31348428/SRR31348474
*Swietenia macrophylla*	J.W. Stead 182 (K)	Guatemala	SRR31348440/SRR31348484
*Swietenia macrophylla*	T.D. Pennington, P. E. Owen & R. Zuniga 13674 (K)	Honduras	SRR31348434/SRR31348481
*Swietenia macrophylla*	R. Alvarez 237 (K)	Mexico	SRR31348444/NA
*Swietenia macrophylla*	T.D. Pennington & J. Sarukhan K. 9629 (K)	Mexico	SRR31348443/SRR31348486
*Swietenia macrophylla*	E. Martinez S. & C. H. Ramos 26392 (K)	Mexico	SRR31348433/SRR31348480
*Swietenia macrophylla*	T.D. Pennington & J. Sarukhan K. 9411 (K)	Mexico	SRR31348442/SRR31348485
*Swietenia macrophylla*	P.P. Moreno 24029 (K)	Nicaragua	SRR31348435/SRR31348482
*Swietenia macrophylla*	T.D. Pennington, A. Daza, J. Revilla 17369 (K)	Peru	SRR31348427/SRR31348473
*Swietenia macrophylla*	s.coll. K004794033 T.7010 (K)	Trinidad and Tobago	SRR31348432/SRR31348479
*Swietenia macrophylla*	T. Carrillo CH. 29 (K)	Venezuela	SRR31348429/SRR31348475
*Swietenia mahagoni*	P. Wilson 7434 (K)	Bahamas	SRR31348420/SRR31348612
*Swietenia mahagoni*	A. G. Large 1942‐27 (K)	Barbados	SRR31348419/SRR31348611
*Swietenia mahagoni*	K004794014 Encarnacion, W. s.n. (K)	Dominican Republic	SRR31348422/SRR31348469
*Swietenia mahagoni*	Hawthorn W., Cable S., & Wise R. 458 (K)	Grenada	SRR31348371/SRR31348609
*Swietenia mahagoni*	W.M. Harris 10821 (K)	Jamaica	SRR31348423/SRR31348470
*Swietenia mahagoni*	E. Freid 08‐166 (K)	Turks and Caicos Islands	SRR31348421/SRR31348614
*Swietenia mahagoni*	C. L. Lundell & Amelia A. Lundell 17549 (K)	USA	SRR31348424/SRR31348471
*Entandrophragma angolense*	Philomena Yarwoah QRRL418 (WFID)	Liberia	SRR31348367/SRR31348532
*Entandrophragma angolense*	Philomena Yarwoah UWLV282 (WFID)	Liberia	SRR31348408/SRR31348529
*Khaya ivorensis*	Bouka Gaël FHEL563 (WFID)	Republic of the Congo	SRR31348461/SRR31348508
*Swietenia macrophylla*	Fabiola Lopez KXYH120 (WFID)	Mexico	SRR31348445/SRR31348487

^a^The voucher column contains the collector(s), collection number and specimen location (K: Kew Herbarium; WFID: World Forest ID collection at the Kew Jodrell Laboratory, Richmond, United Kingdom). The last column lists the Sequence Read Archive accession numbers for the target sequence capture (Angiosperms353) and genome skimming datasets (NA indicates that there was no skimming data generated for the sample due to limited DNA library availability). Further details on sequencing protocols and sequencing success are provided in Appendix S2.
