# Supplementary material for: Leveraging target enrichment and genome skimming (Hyb‐Seq) of herbarium collections to unlock timber DNA barcoding
Source: Appl Plant Sci. 2026 Jun 12;14(3):e70063. doi: 10.1002/aps3.70063 (PMC13287967; doi:10.1002/aps3.70063)
Supplement: Supplementary file 1 — Appendix S1: Supplementary methods including wood DNA extraction protocol. [file APS3-14-e70063-s003.docx]

**Appendix S1.** Supplementary methods including wood DNA extraction protocol.

**Taxon sampling**

Leaf tissue was sampled from herbarium specimens mainly hosted at Kew herbarium (K), except for a few that were obtained from World Forest ID collections ([https://worldforestid.org](https://worldforestid.org/)). All species of *Entandrophragma*, *Khaya*, *Lovoa*, and *Swietenia* were sampled except *K. madagascariensis*, a Madagascar endemic. Specimens tentatively identified as *K. anthotheca* were sampled from K and data were generated from them but excluded from the species trees presented in the main results because the different samples were not recovered as monophyletic in preliminary analyses. This is due to the complex taxonomy of this species, which makes it difficult to ascertain if samples previously identified as *K. anthotheca* indeed represent this species or other described (e.g., *K. nyasica*) or yet undescribed species (Bouka et al., 2022). The raw data from samples originally assigned to this species were deposited in the Sequence Read Archive (SRA) of the National Center for Biotechnology Information (NCBI; SRA, 2025) under *Khaya sp.* (Appendix S2), and their sequences have been kept (as *Khaya* cf. *anthotheca*) in the reference alignments of the candidate barcodes tested here as they may be useful to identify samples potentially belonging to this species.

To perform wood identification tests using Hyb-Seq, wood tissue samples representing four *Entandrophragma*, one *Lovoa*, two *Khaya*, and one *Swietenia* species were obtained from World Forest ID. These samples were identified at the species level at the time of collection by a botanist. When possible, heartwood and sapwood were sampled from the same accession, for a total of 9 heartwood and 20 sapwood samples. Four samples of processed (glued) heartwood obtained from a company and tentatively identified as *Entandrophragma sp.* were added to the study to explore the performance of Hyb-Seq on transformed wood. All samples are listed in Appendix S2 with their geographic origin and voucher information.

The PCR amplification and Sanger sequencing of the candidate barcodes (see below) were tested on 19 wood DNA extracts. These included 9 heartwood DNAs from *E. cylindricum* (3 samples from 1 collection), *E. candollei* (1 sample), *K. ivorensis* (1 sample), *K. senegalensis* (2 samples from 2 different collections), and *S. macrophylla* (2 samples from 1 collection), and 10 sapwood DNAs from *E. cylindricum* (2 samples from 1 collection), *E. candollei* (2 samples from 1 collection), *K. senegalensis* (3 samples from 1 collection), *S. macrophylla* (2 samples from 1 collection), and *L. trichilioides* (1 sample). Heartwood and sapwood samples available for the same species (i.e., for *E. cylindricum*, *E. candollei*, *K. senegalensis*, and *S. macrophylla*) always came from the same collection. These samples are listed in Appendix S3.

**DNA extraction**

DNA extraction from herbarium leaf material was performed following a modification of the cetyltrimethylammonium bromide method (Doyle and Doyle, 1987) as described in Brewer et al. (2019). For wood tissue, further modifications of this protocol were sometimes applied depending on the sample, as indicated in Appendices S2 and S3. A major difference with leaf tissue was that wood samples were cut in 0.5 mm^2^ chips and grinded using a metal jar and metal beads before being incubated in the CTAB buffer. The full DNA extraction protocol for wood samples is described below. DNA concentration was measured with a Quantus fluorometer (Promega UK, Southampton, United Kingdom) and DNA size was measured on an Agilent 4200 TapeStation (Agilent Technologies, Stockport, United Kingdom).

*Wood DNA Extraction Protocol*

The protocol is mainly based on Doyle and Doyle (1987), Inglis et al. (2018), and Lu et al. (2020), with adaptations by L. Csiba, S. Bellot and C. Quintero-Berns (RBG Kew). Another lysis buffer containing boric acid (BoTAB; Lowe et al., 2015) was experimented on a few samples (Appendix S3).

*1. Sample preparation and grinding*

1a. Cut or use chisel to break down the wood tissue in small (5 mm) chips, and put them into a stainless steel mill jar for grinding

1b. Put two metal beads in the jar and put all at -80°C if not proceeding quickly to step 1d. If proceeding to 1d after freezing, avoid letting the chips and jar warm to room temperature.

1c. Set up as many labelled 2 mL microcentrifuge tubes as needed depending on the quantity of chips.

1d. Grind the wood chips into a fine powder, for instance by grinding 4 times 6 min at 25 Hz. Adjust the time or number of grinding rounds until powder is obtained. Proceed immediately to the next step.

1e. Using a clean spatula, spoon ca. 300 mg (about a third of a 2 mL tube) of wood powder from the jar into a labelled tube and proceed immediately to the next step.

*2. [Optional] Cleaning*

2a. Add 1500 µL Sorbitol Buffer, 0.35 M + 15 µL Mercaptoethanol

2b. Centrifuge at 5000 rcf for 5 min

2c. Discard supernatant

2d. Repeat steps 2a to 2c

*3. Sample lysis with CTAB*

3a. Warm [745 µl × (number of samples + 2)] of CTAB in 55°C water bath / oven

3b. Just before proceeding with 3c, add [3µl × (number of samples + 2)] of 2-mercaptoethanol to the heated CTAB: this is the extraction buffer

3c. Add 750 µL of this extraction buffer to each tube containing wood powder

3d. Vortex to make sure the CTAB is in contact with all the powder, dissolving any clumps

3e. Incubate tubes at 55°C for 5 h – shaking constantly and/or vortexing regularly

3f. [Optional] 30 min before the end of the incubation, add 15 µL of proteinase K originally prepared at a concentration of 20 mg/mL and vortex

*4.   SEVAG Addition*

4a. Transfer 750 µL of SEVAG to each tube. Close tubes tightly and vortex thoroughly

4b. Agitate tubes horizontally on an orbital shaker for 10 min

4c. Centrifuge tubes at 13,000 rpm for 15 min

4d. Transfer the supernatant to a new labelled tube

4e. Repeat steps above

*5.   DNA Precipitation*

5a. Add equal volume of chilled isopropanol to each tube

5b. [Optional] Add 65 uL (i.e., ca. 1/10^th^ of the volume already in the tube) of sodium acetate (NaOAc 3M pH 5.2) + 10 µL of glycogen

5c. Ensure lids are firmly closed and gently invert tubes several times

5d. Keep in the freezer (-20°C) at least 1 h and overnight if convenient

*6.   DNA Elution and Resuspension*

6a. Centrifuge sample tubes at 13,000 rpm for 20 min

6b. Discard the supernatant in a waste container

6c. Add 750 µL of 70% ethanol to each tube; vortex or flick to detach the pellet from tube

6d. Centrifuge tubes at 13,000 rpm for 5 min

6e. Discard supernatant, being careful to not remove DNA pellet

6f. Repeat ethanol wash (steps 6c–6e)

6g. Open the tubes and allow the pellet to dry completely until no ethanol remains

6h. Heat an aliquot of molecular-grade water in 65°C water bath / oven

6i. Dissolve the ethanol pellet in 50 µL of water (increase to 75 µL or 100 µL if necessary due to viscosity of the DNA)

7. *[Optional]* *Cleaning*

This is not typically done because it can result in significant loss of DNA, but should be considered if the DNA extract is dark brown or black and/or viscous and/or if the final DNA pellet is dark, shiny, and does not easily come off the tube wall at the previous step. Use SPRI beads, not columns.

*Solutions used in the wood DNA extraction protocol*

Sorbitol Buffer 0.35 M (from Inglis et al., 2018)

- 100 mM Tris-HCl pH 8.0

- 0.35 M Sorbitol

- 5 mM EDTA pH 8.0

- 1% (w/v) Polyvinylpyrrolidone (average molecular weight 40,000; PVP-40)

2X CTAB (cetyltrimethylammonium bromide)

- 100 mM Trizma TRIS base, NH2C(CH2OH), C4H11NO3, FW 121.1

100 mL/L 1 M pH 8.0 stock solution

- 20 mM EDTA, C10H14N2O8Na2·2H2O, FW 372.2

80 mL/L 0.25 M pH 8.0 stock solution

- 1.4 M Sodium Chloride, NaCl, FW 58.44, 81.82 g/L

- 2 % (w/v) CTAB, C19H42BrN, FW 364, 20.00 g/L

- 2 % (w/v) PVP, Av.Mol.Wt. 40,000 / Av.Mol.Wt. 36,000, 20.00 g/L

- Molecular-grade H_2_O up to 1000 mL

SEVAG

- 24 units Chloroform, Trichloromethane, CHCl3, FW 119.4

- 1 unit Isoamyl alcohol, 3-Methyl butanol, (CH3)2CHCH2CH2OH, FW 88.15

**Target sequence capture and Illumina sequencing**

DNA library preparation and Illumina sequencing were performed on the DNA from all samples to be included in the reference dataset (i.e., all the DNA samples extracted from leaf tissue, Appendix S2) and on a subset of the DNA samples obtained from wood tissue (Appendix S2). DNA samples with a high proportion of DNA fragments longer than 500 bp were sonicated using a Covaris ME220 Focused-ultrasonicator (Covaris, Brighton, United Kingdom), and DNA library preparation was performed using a NEBNext Ultra II library kit (New England BioLabs, Hitchin, United Kingdom). Depending on DNA quality, the size selection step included in the protocol was performed or not, the kit’s Adapter solution was diluted or not, and a different number of PCR cycles was performed (details are provided for each sample in Appendix S2). Libraries were dual-indexed using the NEBNext Multiplex Oligos for Illumina (New England BioLabs).

DNA libraries were pooled to reach approximately equal molarities of all libraries in a given pool. Libraries from wood DNA were pooled separately. This resulted in 10 pools of 14 to 26 libraries. In order to increase the likelihood of obtaining plastid genome and ITS data for the reference samples, a separate “genome skimming” pool was made by pooling together 2 μL from each pool made from DNA obtained from leaf tissue, while the rest of the pools were subjected to target sequence capture. Pools made from DNA obtained from wood tissue were entirely subjected to target sequence capture as their low DNA content did not allow a fraction to be kept for genome skimming. Target sequence capture was done by using the Angiosperms353 RNA bait kit (Johnson et al., 2019) manufactured by Daicel Arbor Biosciences (Ann Arbor, Michigan, USA), following their “Standard” protocol (<https://arborbiosci.com/wp-content/uploads/2023/06/myBaits_Manual_v5.03.pdf>). The hybridization step lasted 24 h at 62°C for the leaf DNA pools and 27 h at 63°C for wood DNA pools (because longer incubation times can help enrichment of low-quality DNA while higher temperatures can help reduce off-target DNA capture). Hybridization was followed by PCR amplification (16–20 cycles depending on the pool). The resulting pools enriched in the 353 genes targeted by the Angiosperms353 baits were then pooled together for Illumina sequencing, keeping DNA from leaf and wood tissue in two separate “enriched” pools. The “skimming” leaf DNA pool and the two “enriched” leaf and wood DNA pools were separately sequenced on an Illumina NovaSeq X platform at Macrogen (Seoul, Korea) to generate 150-bp-long paired-end sequencing reads for each sample.

**Illumina data processing and recovery of target DNA regions**

Reads obtained from target capture sequencing and genome skimming were pooled together. Sequence data quality was assessed using FASTQC version 0.11.9 (Andrews, 2010). Illumina sequencing adapters were removed using Trimmomatic v.0.39 (Bolger et al., 2014) in paired-end palindrome mode with one seed mismatch allowed, palindrome and simple clips thresholds of 30 and 7 respectively, a minimum adapter length of 2 bp, and keeping both reads. Bases with low quality at the end of the reads were then removed using a sliding window (“SLIDINGWINDOW” parameter) of 4 bp and a minimum Phred quality threshold of 30, while bases of low quality at the beginning of the reads were removed using the “LEADING” parameter and the same minimum quality threshold. Reads shorter than 40 bp were discarded.

Reads remaining after the above quality filtering were first analyzed using HybPiper v.2 (Johnson et al., 2016) to recover and assemble the 353 regions targeted by the Angiosperms353 bait kit and 177 regions representing the full chloroplast genome. We used the default Angiosperms353 reference sequences available at <https://github.com/mossmatters/Angiosperms353/blob/master/Angiosperms353_targetSequences.fasta>, complemented with 78 coding sequences, 95 intergenic spacers, and four ribosomal RNA sequences from the plastome of *Swietenia mahagoni* (GenBank accession NC_040009). Many genes (between 0 and 305 depending on the sample) were flagged as potentially paralogous by HybPiper, so we used CAPTUS v.1 (Raza et al., 2023; Ortiz et al., 2024) to perform the final gene assembly, which allowed us to test and compare two approaches for handling paralogy. Indeed, CAPTUS enables a flexible use of the multiple copies (potential paralogs) assembled for a given gene: they can either be all used in downstream phylogenetic analyses (hereafter this approach is referred to as the paralog-inclusive [PI] approach), or one of the copies can be selected for downstream analyses based on its similarity to the reference sequences (hereafter paralog-exclusive [PE] approach). To explore the impact of potential paralogs on phylogenetic inferences, we followed both approaches and compared the resulting trees. Two new files of reference sequences were created for the CAPTUS analyses. The first file comprised only nuclear protein-coding regions, including the above-mentioned Angiosperms353 references and Angiosperms353 regions assembled from four high-quality samples (one per genus) during our HybPiper analysis. This reference file was purified from sequences of low complexity or with internal stop codons using the check_targetfile and fix_targetfile commands in HybPiper v.2. The second file comprised the plastome regions mentioned above and published sequences of the ITS region from 13 species belonging to the four focus genera. Reference files are available at <https://github.com/sidonieB/Bellot_al_Meliaceae_DNA_barcoding>. CAPTUS was ran on both reference files, selecting the “GE” and “MA” options to allow retrieval of non-targeted areas (e.g., introns).

**Sequence analysis and phylogenetic inferences**

For each nuclear (including ITS) and plastid region, the sequences of all samples (including potential paralogs in the PI analysis) were aligned using MAFFT v.7 (Katoh and Standley, 2013) and the alignments were trimmed from gappy columns using ClipKIT (Steenwyk et al., 2020) inside the CAPTUS pipeline. Trimmed alignments were then further cleaned with CIAlign v.1.1.0 (Tumescheit et al., 2022) to remove divergent, likely spurious sequences (with option “--remove_divergent_minperc 85” and option “retain-str” to prevent the outgroup to be discarded due to higher divergence) and then with TAPER v.1.0 (Zhang et al., 2021) using default settings to remove small misaligned stretches. Clean nuclear alignments with a median ungapped sequence length <300 bp were discarded as they would likely not contain enough phylogenetic signal to inform gene tree inferences, while all plastid alignments were kept as they would be concatenated before inferring a plastome tree.

For each nuclear alignment, a gene tree was estimated with IQ-TREE v.1.6.12 (Minh et al., 2020), selecting the best model of nucleotide substitution with ModelFinder (Kalyaanamoorthy et al., 2017), and performing 1000 ultrafast bootstrap replicates. The nuclear gene trees resulting from the PE approach (except the ITS tree, which was kept separate) were analyzed using Weighted Astral v.1.16.3.4 (Zhang et al., 2018; Zhang and Mirarab, 2022) to generate a species tree. The nuclear gene trees resulting from the PI approach (and therefore often comprising multiple gene copies for a given sample) were decomposed into single-copy gene trees using DISCO v.1.4 (Willson et al., 2022), and the latter were then analyzed with weighted ASTRAL to generate a species tree where each sample was represented only once. All plastid alignments were concatenated using AMAS (Borowiec, 2016), and a phylogenetic tree was inferred based on the concatenated alignment using IQ-TREE with 1000 bootstrap replicates. The best partition scheme and corresponding best models of nucleotide substitutions were estimated through IQ-TREE’s ModelFinder and TESTMERGE option (Chernomor et al., 2016).

Before being analyzed for the gene barcoding potential assessment (described in the Methods section of the article), gene trees were rooted on *Schmardaea* or, when not present in the tree, on *Lovoa*, using the pxrr program of the Phyx toolkit (Brown et al., 2017). Gene trees that could not be rooted on either of the two genera were discarded from barcoding assessment analyses as lacking one of the focus genera would make them suboptimal for DNA barcoding in our context.

**PCR and sequencing of barcodes from wood DNA**

Primers used to amplify the candidate barcodes are provided in Appendix S7. PCR of the ITS1 region used primers 17SE and ITS-2, described in Sun et al. (1994) and White et al. (1990), respectively, and PCR of the plastid intergenic spacer *trnL-trnF* used primers e and f (described in Taberlet et al., 1991), also provided in Appendix S7. High-molecular-weight DNAs from leaf tissue of *E. angolense* and *K. senegalensis* that had been successfully used to generate reference data (i.e., samples EA6 and KS126; Appendix S2) were used as positive controls, while water was used as negative control. PCR was done by mixing 2 μL of the sample DNA with 8.5 μL of water, 10 μL of TBT (5X), 2.5 μL of DMSO, 1 μL of each primer (10 μM), and 25 μL of Taq polymerase (2X ‘Dream Taq’ [Thermo Fisher Scientific, Waltham, Massachusetts, USA], 4.0 mM MgCL_2_). The mix was then incubated in a thermocycler for 2 min at 94°C + 28 × (1 min at 94°C + 1 min at 52°C + 1 min at 72°C) + 7 min at 72°C. PCR products were analyzed by electrophoresis in a 1% agarose gel with SYBR Safe dye (Invitrogen, Waltham, Massachusetts, USA) dye and photographed using a UVP GelStudio (Analytikjena, Jena, Germany). PCR products that showed clear bands in the electrophoresis were purified using the Macherey-Nagel NucleoSpin Purification Kit (Düren, Germany). Clean products were sequenced using a 3730xl DNA Analyzer (Applied Biosystems, Waltham, Massachusetts, USA).

**Barcode sequence analyses and sample identification**

Sequences obtained by Sanger sequencing were corrected as needed when the base call did not match the peak observed in the chromatograms, overlapping peaks were coded with the corresponding ambiguity code, and sequence ends with unclear or failed base calling were removed. This was done in Geneious Prime 2024 or UGENE (Okonechnikov et al., 2012). When available, the forward and reverse reads were joined into a single sequence. All clean sequences were then checked for non-plant contamination by comparing them with the “Core nucleotide database” of NCBI’s GenBank (GenBank, 2025) using the “blastn” algorithm (Altschul et al., 1990), as implemented in the dedicated online portal (<https://blast.ncbi.nlm.nih.gov/Blast.cgi?PROGRAM=blastn&PAGE_TYPE=BlastSearch&LINK_LOC=blasthome>) but done through Geneious Prime 2024. Sequences that matched plants or that returned no results at all were kept because the candidate barcodes were not yet present in GenBank for the focus genera or even Meliaceae, while four sequences were discarded as they matched bacterial or animal genes. The sequences were then aligned to the reference sequences available for each barcode, using MAFFT v.7 (Katoh and Standley, 2013). When multiple sequences from a DNA sample were available for the same region, a consensus was made (including Ns and ambiguities if there was any gap or conflict between the sequences) so that final alignments contained only one sequence per sample. Gene trees were then generated from each alignment, using IQ-TREE with automatic selection of the nucleotide substitution model and 1000 ultrafast bootstrap replicates (Kalyaanamoorthy et al., 2017; Minh et al., 2020).

**REFERENCES**

Altschul, S. F., W. Gish, W. Miller, E. W. Myers, and D. J. Lipman. 1990. Basic local alignment search tool. *Journal of Molecular Biology* 215: 403–410.

Andrews, S. 2010. FastQC: A Quality Control Tool for High Throughput Sequence Data.  http://www.bioinformatics.babraham.ac.uk/projects/fastqc/ (21 June 2024, date last accessed).

Bolger, A. M., M. Lohse, and B. Usadel. 2014. Trimmomatic: A flexible trimmer for Illumina sequence data. *Bioinformatics* 30: 2114–2120.

Borowiec, M. L. 2016. AMAS: A fast tool for alignment manipulation and computing of summary statistics. *PeerJ* 2016.

Bouka, G. U. D., C. Doumenge, M. R. M. Ekué, K. Daïnou, J. Florence, B. Degen, J. J. Loumeto, et al. 2022. Khaya revisited: Genetic markers and morphological analysis reveal six species in the widespread taxon K. anthotheca. *Taxon* 71: 814–832.

Brewer, G. E., J. J. Clarkson, O. Maurin, A. R. Zuntini, V. Barber, S. Bellot, N. Biggs, et al. 2019. Factors Affecting Targeted Sequencing of 353 Nuclear Genes From Herbarium Specimens Spanning the Diversity of Angiosperms. *Frontiers in Plant Science* 10.

Brown, J. W., J. F. Walker, and S. A. Smith. 2017. Phyx: Phylogenetic tools for unix. *Bioinformatics* 33: 1886–1888.

Chernomor, O., A. Von Haeseler, and B. Q. Minh. 2016. Terrace Aware Data Structure for Phylogenomic Inference from Supermatrices. *Systematic Biology* 65: 997–1008.

Doyle, J. J., and J. L. Doyle. 1987. A rapid DNA isolation procedure for small quantities of fresh leaf tissue. *Phytochemical Bulletin* 19: 11–15.

Inglis PW, Pappas MdCR, Resende LV, Grattapaglia D (2018) Fast and inexpensive protocols for consistent extraction of high quality DNA and RNA from challenging plant and fungal samples for high-throughput SNP genotyping and sequencing applications. PLoS ONE 13(10): e0206085. <https://doi.org/10.1371/journal.pone.0206085>

Johnson, M. G., E. M. Gardner, Y. Liu, R. Medina, B. Goffinet, A. J. Shaw, N. J. C. Zerega, and N. J. Wickett. 2016. HybPiper: Extracting coding sequence and introns for phylogenetics from high‐throughput sequencing reads using target enrichment. *Applications in Plant Sciences* 4.

Johnson, M. G., L. Pokorny, S. Dodsworth, L. R. Botigué, R. S. Cowan, A. Devault, W. L. Eiserhardt, et al. 2019. A Universal Probe Set for Targeted Sequencing of 353 Nuclear Genes from Any Flowering Plant Designed Using k-Medoids Clustering. *Systematic Biology* 68: 594–606.

Kalyaanamoorthy, S., B. Minh, T. Wong, A. von Haeseler, and L. S. Jermiin. 2017. ModelFinder: fast model selection for accurate phylogenetic estimates. *Nature Methods*: 587–589.

Katoh, K., and D. M. Standley. 2013. MAFFT multiple sequence alignment software version 7: Improvements in performance and usability. *Molecular Biology and Evolution* 30: 772–780.

Lowe, A. J., Jardine, D. I., Cross, H. B., Degen, B., Schindler, L., Hoeltken, A. M. (2015). A method of extracting plant nucleic acids from lignified plant tissue. International Patent Number WO/2015/070279.

Lu, Y., Jiao, L., He, T., Zhang, Y., Jiang, X. and Yin, Y., 2020. An optimized DNA extraction protocol for wood DNA barcoding of *Pterocarpus erinaceus*. IAWA Journal, 41(4), pp.644-659.

Minh, B. Q., H. A. Schmidt, O. Chernomor, D. Schrempf, M. D. Woodhams, A. Von Haeseler, R. Lanfear, and E. Teeling. 2020. IQ-TREE 2: New Models and Efficient Methods for Phylogenetic Inference in the Genomic Era. *Molecular Biology and Evolution* 37: 1530–1534.

Okonechnikov, K., O. Golosova, M. Fursov, A. Varlamov, Y. Vaskin, I. Efremov, O. G. German Grehov, et al. 2012. Unipro UGENE: A unified bioinformatics toolkit. *Bioinformatics* 28: 1166–1167.

Ortiz, E. M., A. Höwener, G. Shigita, M. Raza, O. Maurin, A. Zuntini, F. Forest, et al. 2024. A novel phylogenomics pipeline reveals complex patterns of reticulate evolution in Cucurbitales. *BioRxiv*.

Raza, M., E. M. Ortiz, L. Schwung, G. Shigita, and H. Schaefer. 2023. Resolving the phylogeny of Thladiantha (Cucurbitaceae) with three different target capture pipelines. *BMC Ecology and Evolution* 23.

SRA. 2025. Sequence Read Archive (SRA) [Internet]. Bethesda (MD): National Library of Medicine (US), National Center for Biotechnology Information; 2009 - [cited 2025 01 13]. Available from: https://www.ncbi.nlm.nih.gov/sra/.

Steenwyk, J. L., T. J. Buida, Y. Li, X. X. Shen, and A. Rokas. 2020. ClipKIT: A multiple sequence alignment trimming software for accurate phylogenomic inference. *PLoS Biology* 18.

Sun, Y., D. Z. Skinner, G. H. Liang, and S. H. Hulbert. 1994. Phylogenetic analysis of Sorghum and related taxa using internal transcribed spacers of nuclear ribosomal DNA. *Theoretical and Applied Genetics* 89: 26–32.

Taberlet, P., L. Gielly, G. Pautou, and J. Bouvet. 1991. Universal primers for amplification of three non-coding regions of chloroplast DNA. *Plant Molecular Biology* 17: 1105–1109.

Tumescheit, C., A. E. Firth, and K. Brown. 2022. CIAlign: A highly customisable command line tool to clean, interpret and visualise multiple sequence alignments. *PeerJ*.

White, T. J., T. Bruns, S. Lee, and J. Taylor. 1990. Amplification and direct sequencing of fungal ribosomal RNA genes for phylogenetics. *In* M. A. Innis, D. H. Gelfand, J. J. Sninsky, and T. J. White [eds.], PCR protocols - a guide to methods and applications, 315–322. Academic Press.

Willson, J., M. S. Roddur, B. Liu, P. Zaharias, and T. Warnow. 2022. DISCO: Species Tree Inference using Multicopy Gene Family Tree Decomposition. *Systematic Biology* 71: 610–629.

Zhang, C., and S. Mirarab. 2022. Weighting by Gene Tree Uncertainty Improves Accuracy of Quartet-based Species Trees. *Molecular Biology and Evolution* 39.

Zhang, C., M. Rabiee, E. Sayyari, and S. Mirarab. 2018. ASTRAL-III: Polynomial time species tree reconstruction from partially resolved gene trees. *BMC Bioinformatics* 19.

Zhang, C., Y. Zhao, E. L. Braun, and S. Mirarab. 2021. TAPER: Pinpointing errors in multiple sequence alignments despite varying rates of evolution. *Methods in Ecology and Evolution* 12: 2145–2158.
