# Supplementary material for: Leveraging target enrichment and genome skimming (Hyb‐Seq) of herbarium collections to unlock timber DNA barcoding
Source: Appl Plant Sci. 2026 Jun 12;14(3):e70063. doi: 10.1002/aps3.70063 (PMC13287967; doi:10.1002/aps3.70063)

DOI 10.1002/aps3.70063

APPENDIX S4. Intra- and interspecific relationships in four Meliaceae genera based on the paralog-inclusive and paralog-exclusive analyses of 343 and 350 nuclear genes, respectively, the analysis of 177 plastome regions, and the analysis of the ITS region. Numbers on branches indicate local posterior probabilities (nuclear genes) or bootstrap support percentages (plastome, ITS). Circles indicate sapwood (yellow) and heartwood (brown) samples.

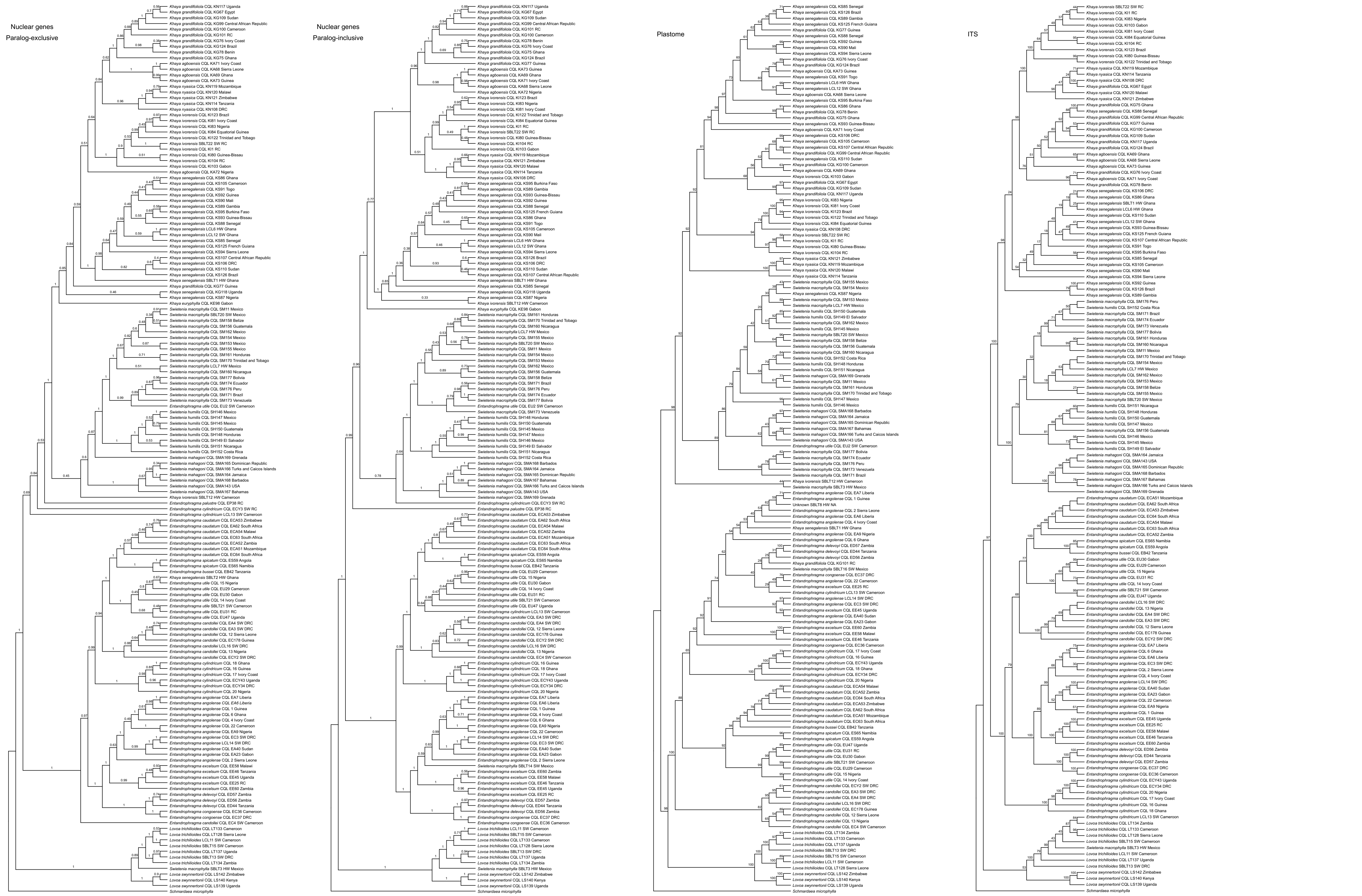

Supplement: Supplementary file 4 — Appendix S4: Intra‐ and interspecific relationships in four Meliaceae genera based on the paralog‐inclusive and paralog‐exclusive analyses of 343 and 350 nuclear genes, respectively, the analysis of 177 plastome regions, and the analysis of the ITS region. [file APS3-14-e70063-s005.pdf]
