# Supplementary material for: Leveraging target enrichment and genome skimming (Hyb‐Seq) of herbarium collections to unlock timber DNA barcoding
Source: Appl Plant Sci. 2026 Jun 12;14(3):e70063. doi: 10.1002/aps3.70063 (PMC13287967; doi:10.1002/aps3.70063)

DOI 10.1002/aps3.70063

APPENDIX S5. Phylogenetic placement accuracy depending on region recovery and genus. Placement categories indicate if the sample was recovered in the right species (Species), the right genus (Genus), or neither (Other). For leaf samples (L), placement is assessed based on the clade in which the sample falls, not considering wood samples. For wood samples (HW: heartwood and SW: sapwood), placement is assessed based on the most closely related leaf (reference) sample, under the rationale that this would be sufficient in a context of DNA barcoding where the whole tree does not need to be perfectly resolved to allow sample identification.

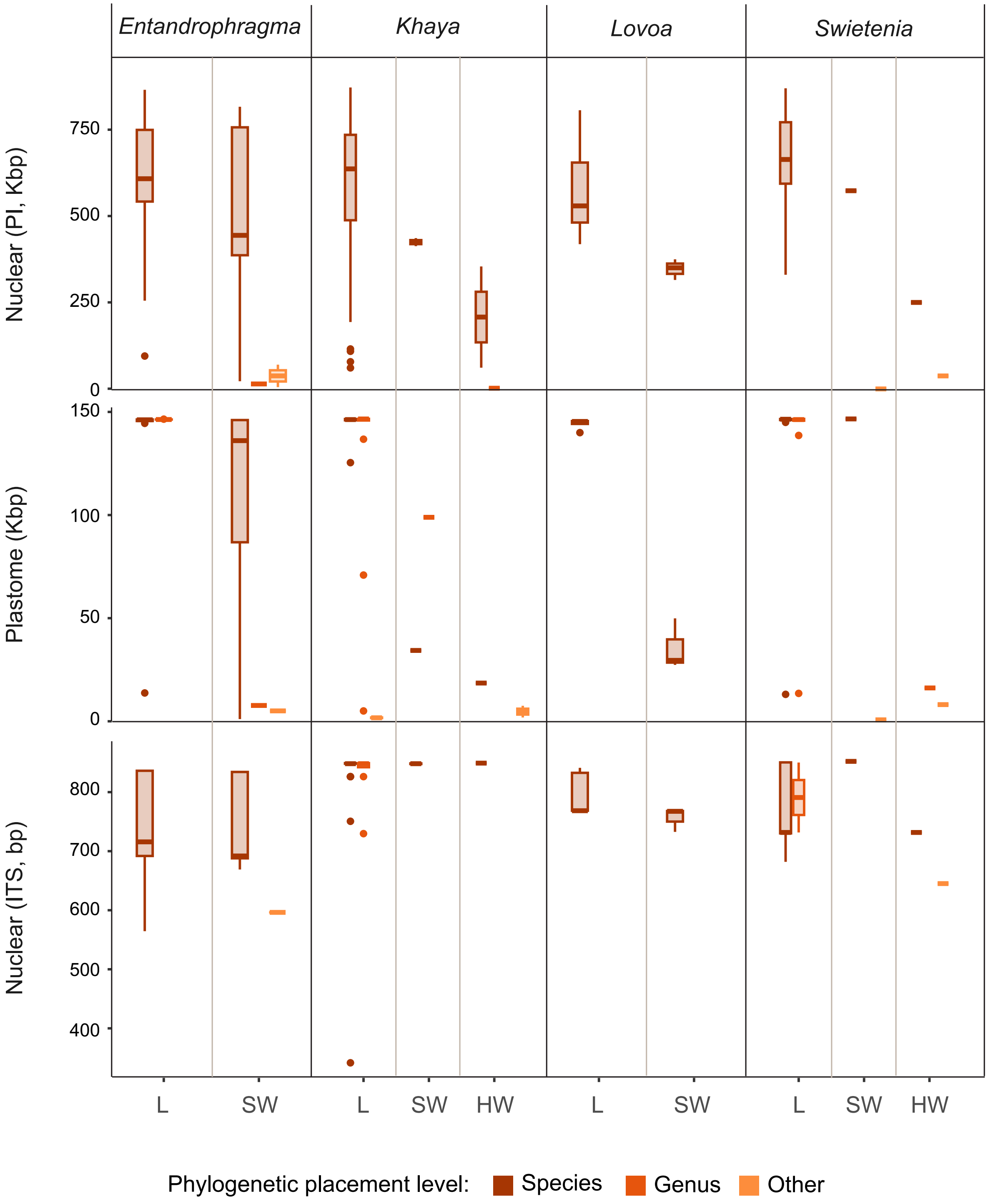

Supplement: Supplementary file 5 — Appendix S5: Phylogenetic placement accuracy depending on region recovery and genus. [file APS3-14-e70063-s008.pdf]
