# Supplementary material for: Leveraging target enrichment and genome skimming (Hyb‐Seq) of herbarium collections to unlock timber DNA barcoding
Source: Appl Plant Sci. 2026 Jun 12;14(3):e70063. doi: 10.1002/aps3.70063 (PMC13287967; doi:10.1002/aps3.70063)

DOI 10.1002/aps3.70063

APPENDIX S6. Schematic representation of the 12 new barcodes and corresponding primers. Arrows represent primers designed in this study. Coordinates indicate the position of the regions in the reference alignments. The size of an individual region is the median length of the sequences in the reference alignments obtained from the target capture sequencing data. Scale is not respected.

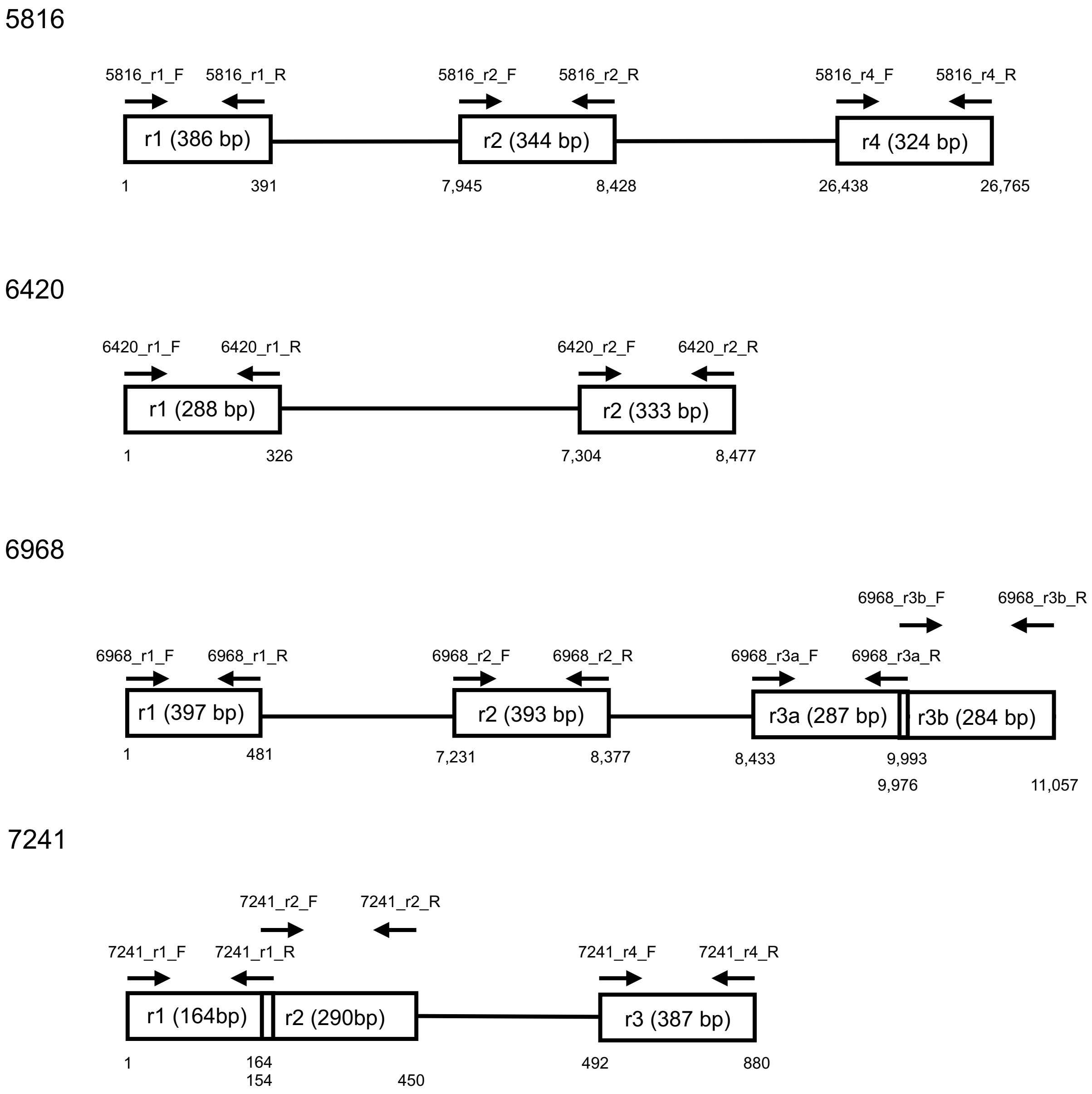

Supplement: Supplementary file 6 — Appendix S6: Schematic representation of the 12 new barcodes and corresponding primers. [file APS3-14-e70063-s011.pdf]
