# Supplementary material for: Leveraging target enrichment and genome skimming (Hyb‐Seq) of herbarium collections to unlock timber DNA barcoding
Source: Appl Plant Sci. 2026 Jun 12;14(3):e70063. doi: 10.1002/aps3.70063 (PMC13287967; doi:10.1002/aps3.70063)

DOI 10.1002/aps3.70063

**APPENDIX S9.** Size of the DNA used for PCR and Sanger sequencing of the barcodes. The top panel is a simulated electrophoresis gel generated by the Tape Station, while the bottom panel displays corresponding graphs for each sample. Sample codes follow Appendix S3. Warning signs indicate concentrations outside of the range expected by the Tape Station so results should be interpreted with caution, but the fragment size distributions can still be assessed as the ladders show peaks where expected.

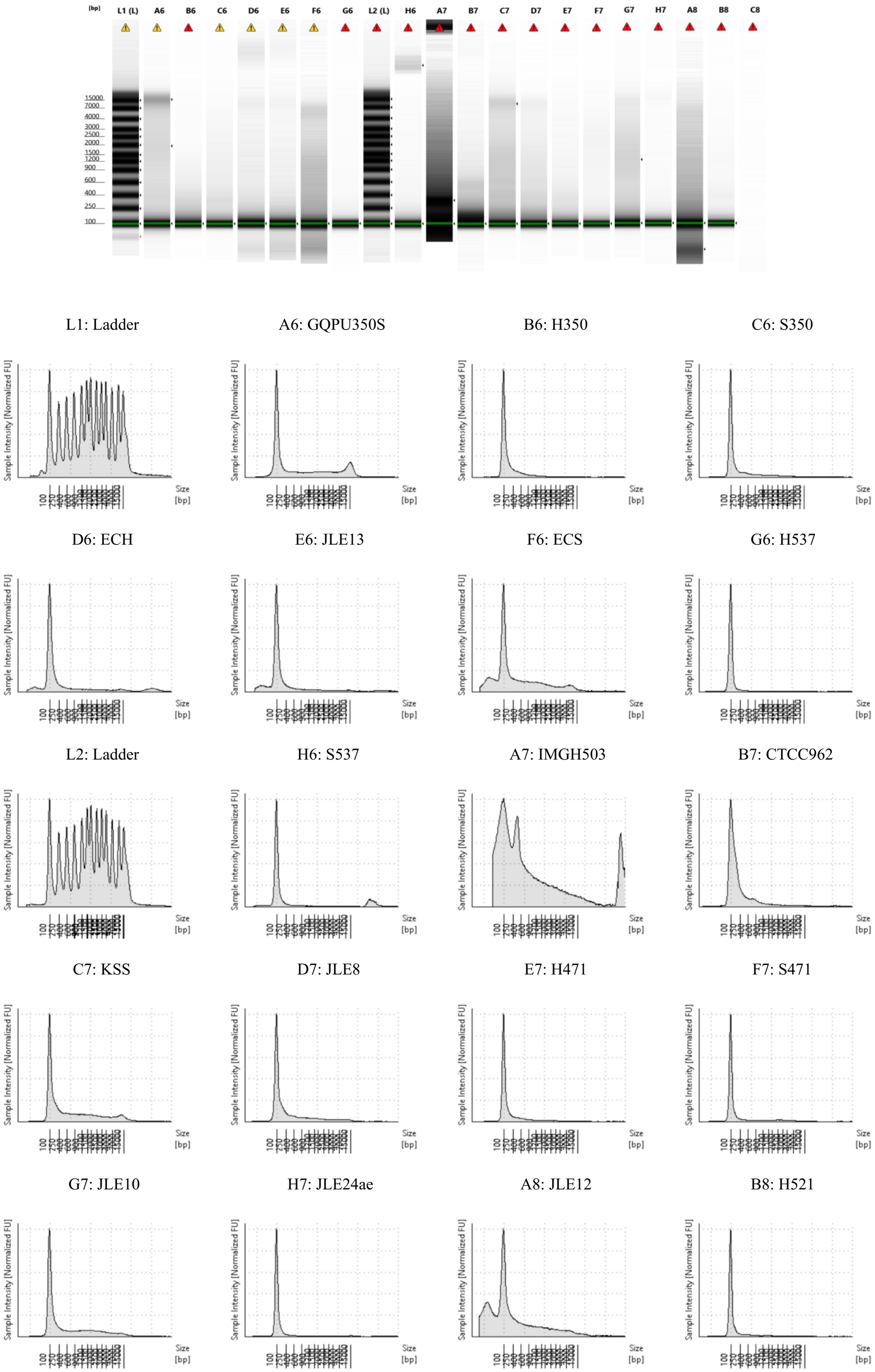

Supplement: Supplementary file 9 — Appendix S9: Size of the DNA used for PCR and Sanger sequencing of the barcodes. [file APS3-14-e70063-s001.pdf]
