# Supplementary figures and images for: Leveraging target enrichment and genome skimming (Hyb‐Seq) of herbarium collections to unlock timber DNA barcoding
Source: Appl Plant Sci. 2026 Jun 12;14(3):e70063. doi: 10.1002/aps3.70063 (PMC13287967; doi:10.1002/aps3.70063)

**APPENDIX S10.** Relationship between DNA quality and PCR and Sanger sequencing results.

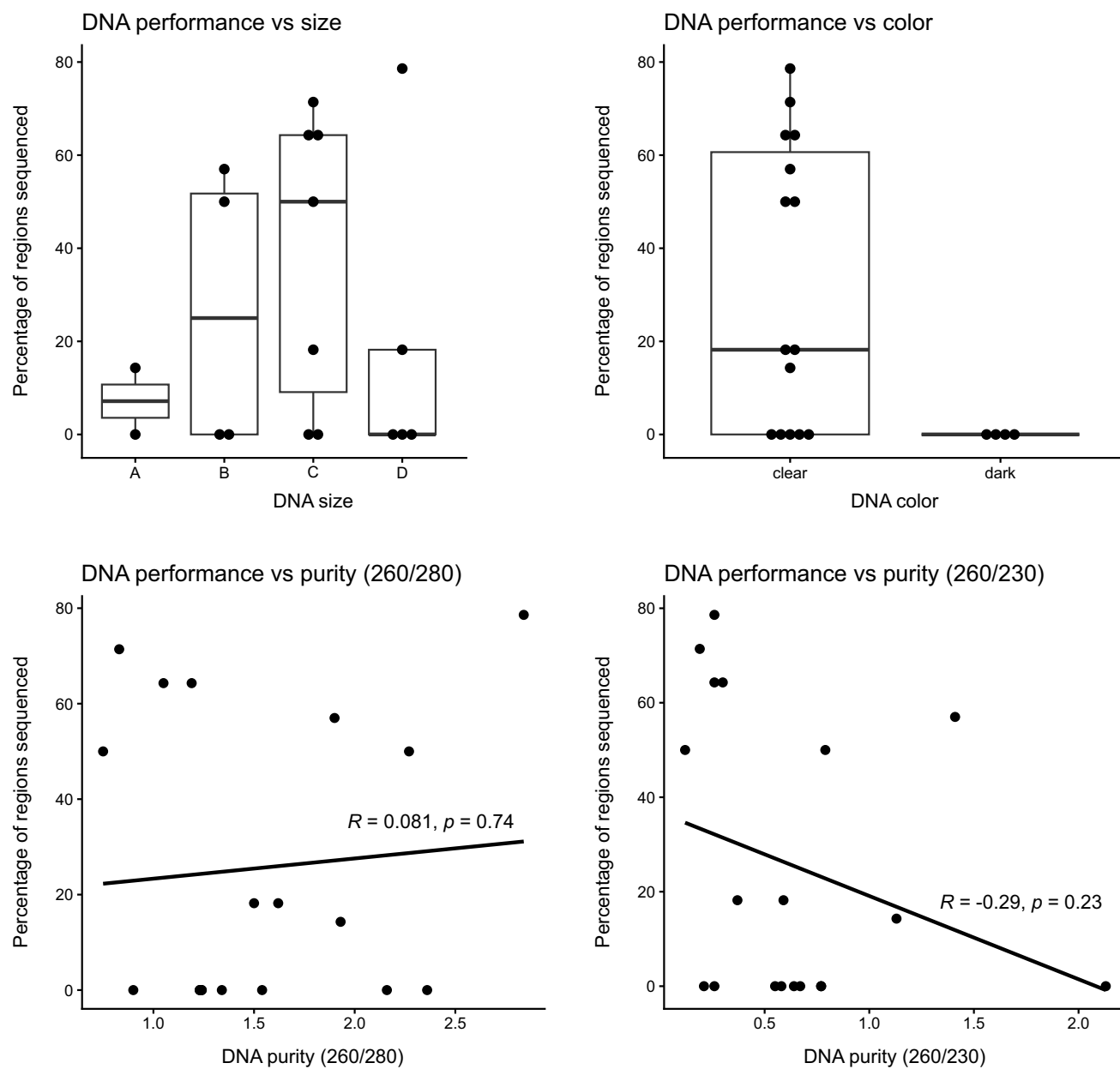

Supplement: Supplementary file 10 — Appendix S10: Relationship between DNA quality and PCR and Sanger sequencing results. [file APS3-14-e70063-s007.pdf]
