# Supplementary material for: Leveraging target enrichment and genome skimming (Hyb‐Seq) of herbarium collections to unlock timber DNA barcoding
Source: Appl Plant Sci. 2026 Jun 12;14(3):e70063. doi: 10.1002/aps3.70063 (PMC13287967; doi:10.1002/aps3.70063)

DOI 10.1002/aps3.70063

APPENDIX S11. Placement of wood samples using the 12 new DNA barcodes, ITS1, and the trnL-trnF region. SW: sapwood, HW: heartwood. Further details on the wood samples are available in Appendix S3. Nodes without bootstrap values result from the random grouping of identical sequences.

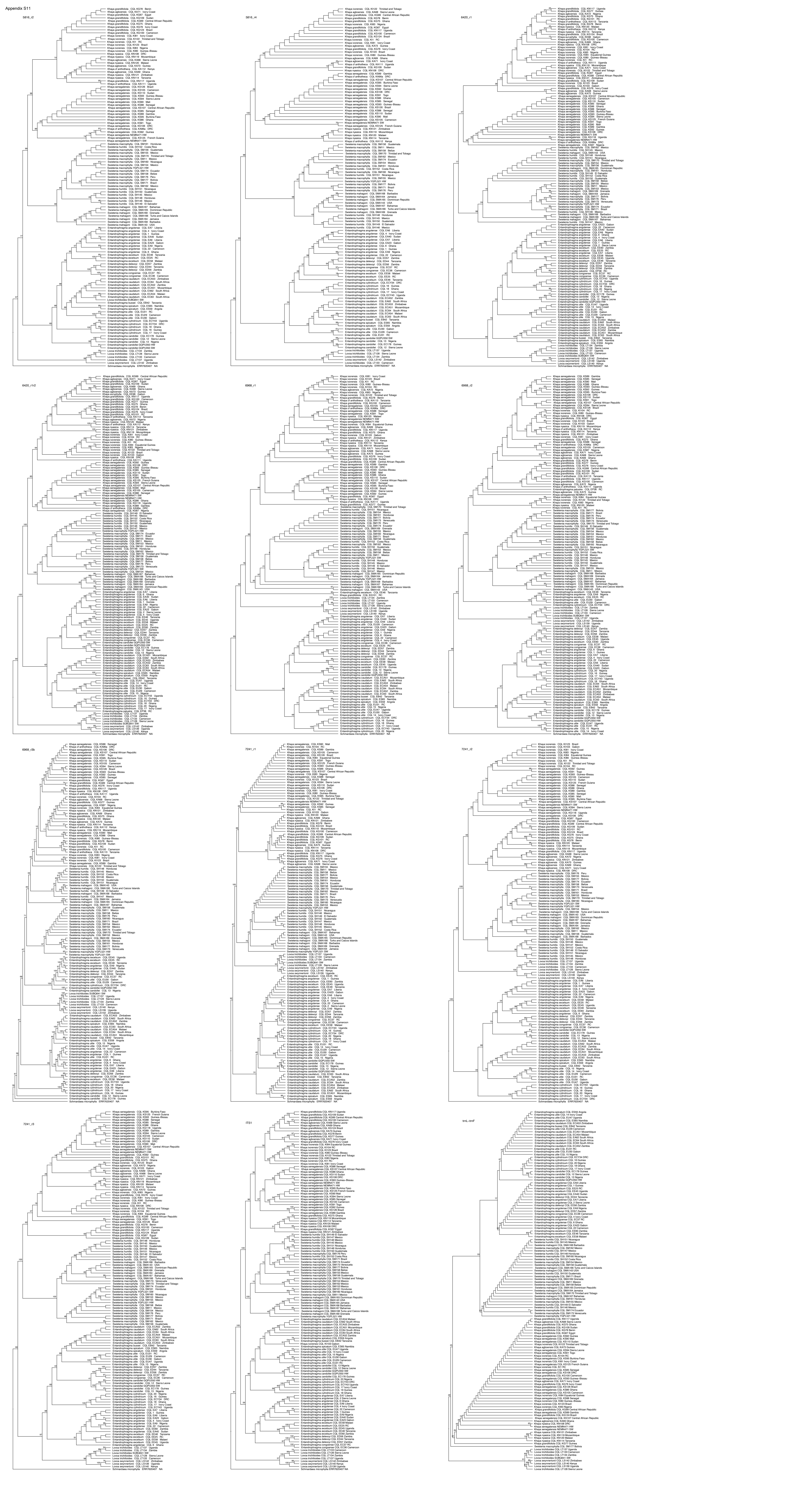

Supplement: Supplementary file 11 — Appendix S11: Placement of wood samples using the 12 new DNA barcodes, ITS1, and the trnL‐trnF region. [file APS3-14-e70063-s009.pdf]
