# Supplementary material for: Leveraging target enrichment and genome skimming (Hyb‐Seq) of herbarium collections to unlock timber DNA barcoding
Source: Appl Plant Sci. 2026 Jun 12;14(3):e70063. doi: 10.1002/aps3.70063 (PMC13287967; doi:10.1002/aps3.70063)

DOI 10.1002/aps3.70063

**APPENDIX S12.** Phylogenetic resolution obtained when combining the best three or six barcodes. Some groups were collapsed for easier visualization. Numbers of individuals per species in a given collapsed group are indicated in brackets. Dots indicate nodes with bootstrap support ≥70%.

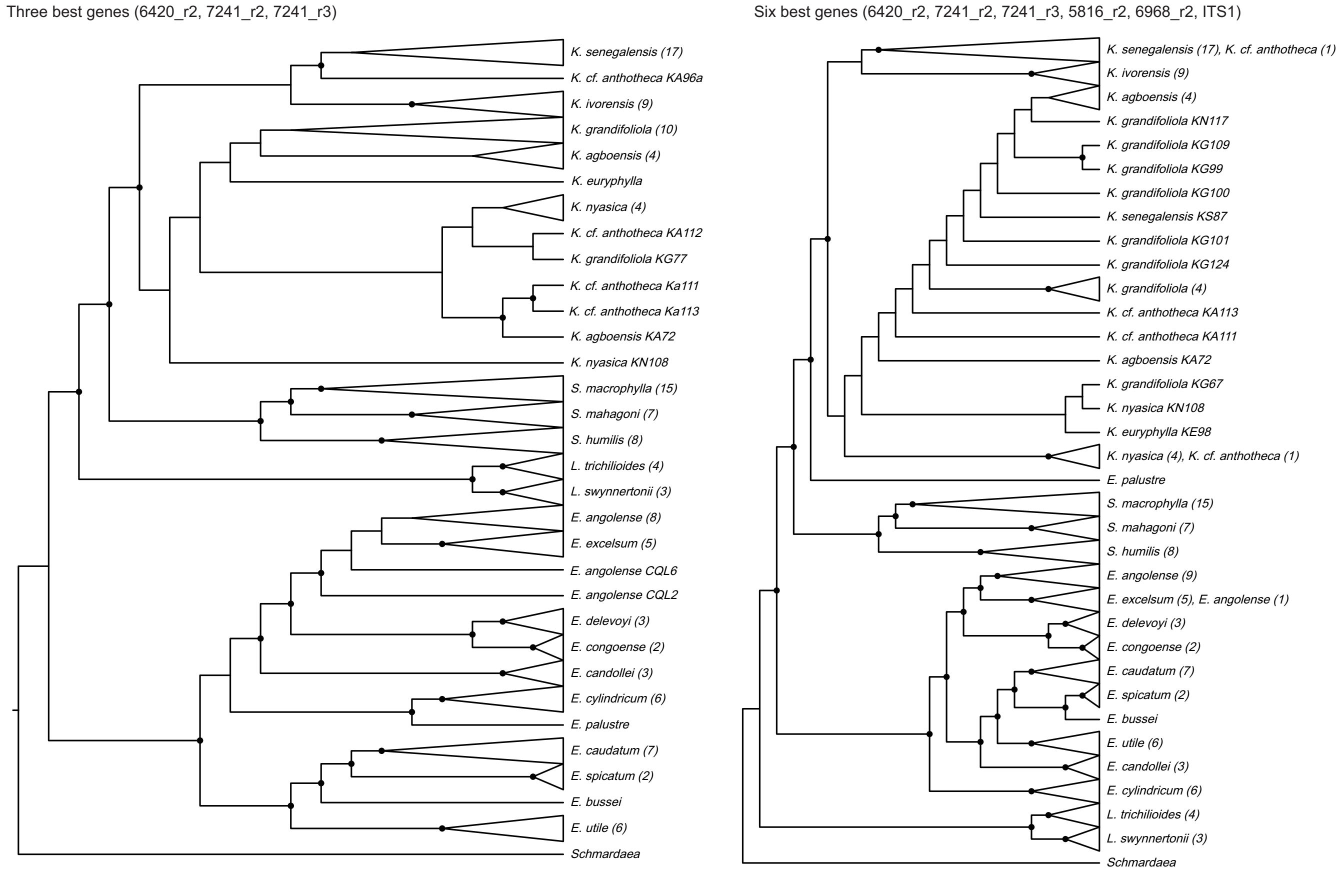

Supplement: Supplementary file 12 — Appendix S12: Phylogenetic resolution obtained when combining the best three or six barcodes. [file APS3-14-e70063-s010.pdf]
